# The Prognostic Significance of Dynamic Monitoring of Minimal Residual Disease Status in Patients with Multiple Myeloma: A Real-World Study

**DOI:** 10.3390/cancers18182958

**Published:** 2026-09-13

**Authors:** Xiaotong Zhang, Xiaozhe Li, Junru Liu, Beihui Huang, Jingli Gu, Meilan Chen, Lifen Kuang, Juan Li

**Affiliations:** Department of Haematology, The First Affiliated Hospital of Sun Yat-sen University, Guangzhou 510080, China

**Keywords:** multiple myeloma, minimal residual disease, autologous stem cell transplantation, survival, real-world study

## Abstract

**Background:** Minimal residual disease (MRD) is an established prognostic factor in multiple myeloma (MM), but the significance of dynamic MRD changes before and after autologous stem cell transplantation (ASCT) remains unclear. **Methods:** We retrospectively analysed 335 newly diagnosed MM patients who underwent upfront ASCT. Overall survival (OS) and progression-free survival (PFS) were estimated using the Kaplan–Meier method and compared using the log-rank test. Cox proportional hazards models were used to estimate hazard ratios (HRs) and 95% confidence interval (CI), adjusting for clinical risk factors. **Results:** Patients were classified as having persistent MRD negativity (*n* = 82, 24.5%), MRD− to MRD+ conversion (*n* = 40, 11.9%), MRD+ to MRD− conversion (*n* = 168, 50.1%), or persistent MRD positivity (*n* = 45, 13.4%). Median OS was not reached: 105, 131, and 87 months for the groups (*p* < 0.001); and PFS was not reached: 50, 71, and 38 months (*p* < 0.001). In multivariable analysis, persistent MRD positivity (HR, 4.762; 95% CI, 2.151–10.546; *p* < 0.001) and MRD− to MRD+ conversion (HR, 3.925; 95% CI, 1.791–8.598; *p* < 0.001) were associated with increased mortality risk compared with persistent MRD negativity, whereas MRD+ to MRD− conversion showed a borderline-significant increase (HR, 1.973; 95% CI, 0.963–4.046; *p* = 0.063). In addition, similar patterns were observed for PFS. In subgroup analyses, late MRD+ to MRD− conversion was associated with inferior PFS (HR, 1.857; 95% CI, 1.136–3.034; *p* = 0.014), whereas MRD− to MRD+ conversion showed no differences in OS or PFS (all *p* > 0.05). Among persistent MRD-positive patients, stable or increasing MRD pattern predicted worse outcomes (HR, 4.397; 95% CI, 1.190–16.240; *p* = 0.026). **Conclusions:** Longitudinal MRD trajectories provided independent prognostic information beyond static MRD assessment in MM. Subgroup analyses further suggest that dynamic MRD assessment might help refine risk stratification after ASCT.

## Simple Summary

Minimal residual disease (MRD) is an established prognostic factor in multiple myeloma (MM), but the significance of dynamic MRD changes before and after autologous stem cell transplantation (ASCT) remains unclear. We retrospectively analyzed 335 patients with newly diagnosed MM who underwent upfront ASCT between 2010 and 2025 and classified them into four longitudinal MRD patterns: persistent negativity, conversion from negativity to positivity, conversion from positivity to negativity, and persistent positivity. Persistent MRD negativity was associated with the most favorable survival, whereas persistent MRD positivity and MRD-negative to MRD-positive conversion identified patients with poor prognosis. Among converters, earlier MRD clearance after ASCT was linked to longer progression-free survival, while the timing of MRD-negative to MRD-positive conversion was not associated with significant survival differences. In persistently MRD-positive patients, a stable or increasing MRD pattern further predicted poor outcomes. These findings suggest that longitudinal MRD assessment may further refine prognostic stratification after ASCT.

## 1. Introduction

Multiple myeloma (MM) is a hematologic malignancy characterized by the clonal accumulation of malignant plasma cells. It accounts for approximately 10% of all hematologic malignancies and is the second most common type of hematological malignancy. Autologous stem cell transplantation (ASCT) remains a standard component of frontline therapy and has substantially improved survival outcomes [1,2]. However, relapse or disease progression after ASCT remains frequent, underscoring the need for close post-transplant monitoring and timely intervention [3,4].

Increasing evidence indicates that minimal residual disease (MRD) assessment was a known surrogate marker for survival in MM [3,5,6,7]. Specifically, previous studies have consistently demonstrated that MRD negativity was associated with prolonged progression-free survival (PFS) and overall survival (OS) [2,8,9], irrespective of treatment regimen, cytogenetic risk profile, or International Staging System (ISS) stage at diagnosis [6,10,11]. Moreover, longer durations of persistent MRD negativity were associated with progressively improved clinical outcomes [2,12,13].

However, MRD status is dynamic, and its prognostic value is substantially enhanced by longitudinal assessments compared with single-time-point evaluation [14,15,16,17,18]. Most previous studies, nevertheless, have assessed MRD at isolated time points or focused on either pre-ASCT or post-ASCT status [19]. Notably, MRD positivity at either the pre- [11] or post-transplant [14] stage has been identified as an independent prognostic factor for disease outcomes [20], although pre-ASCT MRD status was shown to be associated with PFS but not OS [11]. In real-world clinical practice, MRD status frequently evolved during treatment, with more than half of patients experiencing MRD conversion between pre- and post-ASCT assessments [21]. Increasing evidence suggests that quantitative MRD burden might provide additional prognostic information, as deeper MRD responses and progressive reductions in MRD burden were associated with improved clinical outcomes [17,22,23]. Despite these advances, comprehensive evaluations that integrated MRD status, MRD burden, and their dynamics across both treatment phases remained incompletely characterized.

Moreover, because MRD conversion could occur at variable time points after transplantation, a clinically meaningful landmark is needed to distinguish early from late conversion. The 3-month post-ASCT time point has been widely adopted as a key milestone for MRD assessment [13,19]. However, whether MRD conversion occurred before or after this landmark carried distinct prognostic implications and remained unclear.

In this context, we conducted a real-world study of 335 transplant-eligible patients with MM treated at the First Affiliated Hospital of Sun Yat-sen University between 2010 and 2025. This study aimed to (1) evaluate the impact of dynamic MRD patterns on PFS and OS; (2) determine the prognostic relevance of the timing of MRD conversion (early vs. late) among patients who experienced MRD status changes between the pre- and post-ASCT assessments; and (3) assess whether quantitative MRD kinetics provide additional prognostic stratification among patients with persistent MRD positivity.

## 2. Methods

### 2.1. Study Design and Patients

**Figure 1:**
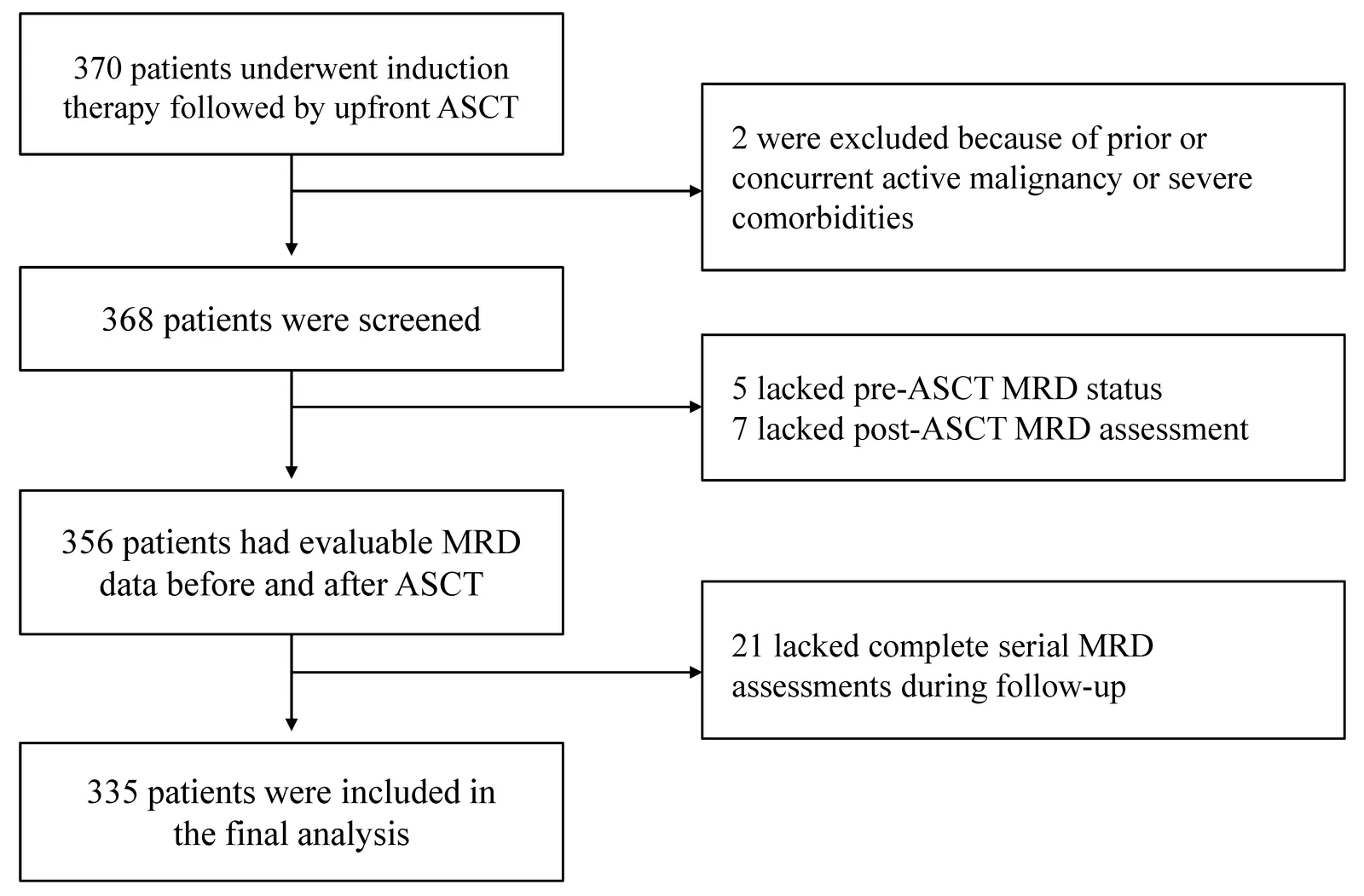
Flow diagram of patient screening and inclusion.

Patients with newly diagnosed multiple myeloma who received induction therapy followed by upfront autologous stem cell transplantation at the First Affiliated Hospital of Sun Yat-sen University between August 2010 and July 2025 were retrospectively screened for eligibility. A total of 370 patients underwent induction therapy followed by upfront ASCT during the study period. Two patients were excluded because of a prior or concurrent active malignancy or severe comorbidities that could substantially affect treatment delivery or survival outcomes. Among the remaining 368 patients, five were excluded because pre-ASCT MRD status was unavailable, and seven were excluded because no evaluable post-ASCT MRD assessment was available. Of the 356 patients with evaluable MRD data both before and after ASCT, 21 were further excluded because the available longitudinal MRD data were insufficient to reliably classify their post-ASCT MRD trajectory. Ultimately, 335 patients were included in the final analysis (Figure 1).

MRD status was assessed longitudinally before and after ASCT. Based on longitudinal changes in MRD status, patients were categorized into four dynamic MRD patterns: persistent MRD negativity (MRD− to MRD−), conversion from MRD-negative to MRD-positive (MRD− to MRD+), MRD-positive to MRD-negative (MRD+ to MRD−), and persistent MRD positivity (MRD+ to MRD+).

All patients met the diagnostic criteria of the International Myeloma Working Group (IMWG). The inclusion criteria were: (1) newly diagnosed multiple myeloma with complete clinical data; (2) receipt of induction therapy followed by ASCT; and (3) availability of evaluable treatment response and serial MRD assessments before and after ASCT. The exclusion criteria were: (1) a prior history of malignancy or the presence of concurrent active malignancies; and (2) severe comorbidities or complications that could substantially affect treatment delivery or survival outcomes. This study followed the principles of the Declaration of Helsinki and was approved by the Ethics Committee of The First Affiliated Hospital of Sun Yat-sen University (IRB [2026]303). The requirement for written informed consent was waived due to the retrospective nature of the study.

### 2.2. MRD Assessment

MRD assessment before ASCT was performed after completion of induction therapy. After transplantation, MRD evaluations were conducted as part of routine clinical practice, scheduled at approximately 3, 6, 9, and 12 months, and every 6 months thereafter. In this real-world retrospective cohort, the actual timing and frequency of post-ASCT MRD assessments varied according to clinical practice and the availability of evaluable bone marrow samples (Supplementary Table S1). Persistent MRD negativity was defined as the absence of flow cytometry-detectable disease across all evaluable MRD assessments performed before and after ASCT, with a minimum sensitivity of 5 × 10^−5^. Persistent MRD positivity was defined as the presence of flow-detectable disease at all corresponding time points using the same sensitivity threshold. MRD+ to MRD− conversion was defined as detectable MRD before ASCT, followed by the first documentation of MRD negativity during post-transplant follow-up. Conversely, MRD− to MRD+ conversion was defined as undetectable MRD before ASCT, followed by the first detection of MRD positivity during longitudinal follow-up. Subsequent MRD fluctuations were not considered for reclassification.

Patients with MRD− to MRD+ conversion and MRD+ to MRD− conversion were further stratified into early (≤3 months after ASCT) and late (>3 months after ASCT) conversion groups. In addition, among patients with persistent MRD positivity, longitudinal MRD kinetics were categorized as decreasing versus stable or increasing MRD burden. MRD burden was quantified as the absolute percentage of phenotypically aberrant plasma cells. Decreasing MRD burden was defined as an overall downward trend in the absolute MRD percentage across serial assessments, whereas stable or increasing MRD burden was defined as a generally stable or upward trend over time.

Bone marrow MRD assessment was performed using multiparameter flow cytometry. The antibody panel included CD38, CD45, CD19, CD20, CD56, CD54, CD138, κ light chain, and λ light chain (BD Biosciences, San Jose, CA, USA). A minimum of 1 × 10^6^ nucleated cells were analyzed per sample. Detection of ≥20 phenotypically aberrant plasma cells was considered MRD-positive, corresponding to a limit of detection of approximately 2 × 10^−5^ to 1 × 10^−5^.

### 2.3. Efficacy Evaluation

Treatment response was assessed according to the International Myeloma Working Group (IMWG) criteria and categorized as complete response (CR), very good partial response (VGPR), partial response (PR), minimal response (MR), stable disease (SD), or progressive disease (PD). Response evaluations were performed after completion of induction therapy, every 3 months during the first year following ASCT and every 6 months thereafter as part of routine clinical follow-up.

### 2.4. Follow-Up

The primary endpoints of the study were PFS and OS. Patients were followed until 26 November 2025. OS was defined as the time from initiation of treatment to death from any cause or last follow-up. PFS was defined as the time from initiation of treatment to documented disease progression or last follow-up.

### 2.5. Statistical Analysis

Categorical variables were presented as frequencies and percentages and compared using the chi-square test, as appropriate. Survival distributions were estimated using the Kaplan–Meier method and compared with the log-rank test. To address potential immortal time bias, a 6-month landmark sensitivity analysis was performed among patients who were alive and progression-free at 6 months after ASCT. MRD groups were defined using assessments available up to the landmark, and survival was calculated from the landmark time point.

Cox proportional hazards regression models were used to evaluate associations between MRD dynamic patterns and survival outcomes in univariate and multivariable analyses. Variables with a *p*-value < 0.10 in univariate analyses, together with potential clinically relevant covariates with established prognostic significance, were included in the multivariable Cox regression models. The number of patients included in each Cox regression analysis is reported in the corresponding tables. Complete-case analysis was performed, and patients with missing values for any covariate included in a given model were excluded from that model. Given the limited extent of missingness, multiple imputation was not performed. In multivariable analyses, OS Cox models were adjusted for Revised International Staging System (R-ISS) staging, hemoglobin, serum lactate dehydrogenase (LDH) level, high-risk cytogenetic abnormalities (HRCAs), cytogenetic abnormalities, and maintenance therapy. PFS Cox models were adjusted for age, R-ISS staging, HRCAs, cytogenetic abnormality, maintenance treatment, and treatment response at ASCT. Hazard ratios (HRs) were reported with corresponding 95% confidence intervals (CIs).

The proportional hazards (PH) assumption was assessed using partial residual-based diagnostics combined with Spearman’s rank correlation analysis. Specifically, the correlations between the partial residuals of each MRD-group dummy variable and survival time were examined. When a significant correlation was identified, an extended Cox model incorporating an interaction term between the corresponding MRD-group dummy variable and time was fitted to further assess whether the effect of that variable changed during follow-up. A statistically significant group-by-time interaction term was considered evidence of a time-dependent effect and a potential violation of the PH assumption.

All statistical analyses were performed using SPSS software (version 27.0; IBM Corp., Armonk, NY, USA) and R software (version 4.5.2). A two-sided *p*-value < 0.05 was considered statistically significant.

## 3. Results

### 3.1. MRD Evaluation and Patient Characteristics

A total of 335 patients undergoing ASCT were included in the analysis (Table 1). Based on dynamic changes in MRD status assessed longitudinally before and after ASCT, patients were classified into four groups: 82 patients (24.5%) with persistent MRD-negative disease, 40 patients (11.9%) who experienced MRD− to MRD+ conversion, 168 patients (50.1%) who converted from MRD+ to MRD−, and 45 patients (13.4%) with persistent MRD-positive disease. The median numbers of documented MRD assessments per patient were 6 (range, 2–10), 8 (range, 3–11), 7 (range, 2–11), and 4 (range, 2–10) in the persistent MRD-negative, MRD− to MRD+, MRD+ to MRD−, and persistent MRD-positive groups, respectively (Supplementary Tables S1 and S2).

The proportion of patients achieving at least CR at the time of ASCT was significantly lower in the persistent MRD-positive group than in the other three MRD dynamic groups (61.0 vs. 47.5% vs. 39.9% vs. 11.1%; *p* < 0.001). In addition, female patients were more frequently represented in the persistent MRD-positive group, with a borderline difference observed among the four groups (*p* = 0.050). In contrast, other baseline characteristics, including age, M-protein type, ISS and R-ISS, cytogenetic abnormalities, HRCAs, induction regimen, maintenance therapy, anti-CD38-based therapy, hemoglobin level, serum LDH level, and transplantation-related complications were well balanced across the four groups (all *p* > 0.05).

To better characterize dynamic changes in MRD, patients with MRD− to MRD+ conversion (*n* = 40) were further stratified into early (≤3 months after ASCT; *n* = 12) and late (>3 months after ASCT; *n* = 28) conversion groups, and a similar early–late stratification was applied to patients with MRD+ to MRD− conversion (*n* = 168; early, *n* = 96; late, *n* = 72) (Table 2). In addition, among patients with persistent MRD positivity (*n* = 45), subgroups were defined according to decreasing (*n* = 20) versus stable or increasing (*n* = 25) MRD burden (Table 3). No significant differences in baseline demographic, disease-related, or treatment-related characteristics were observed across any of these subgroup analyses (all *p* > 0.05).

### 3.2. Overall Survival According to MRD Dynamic Patterns

**Figure 2:**
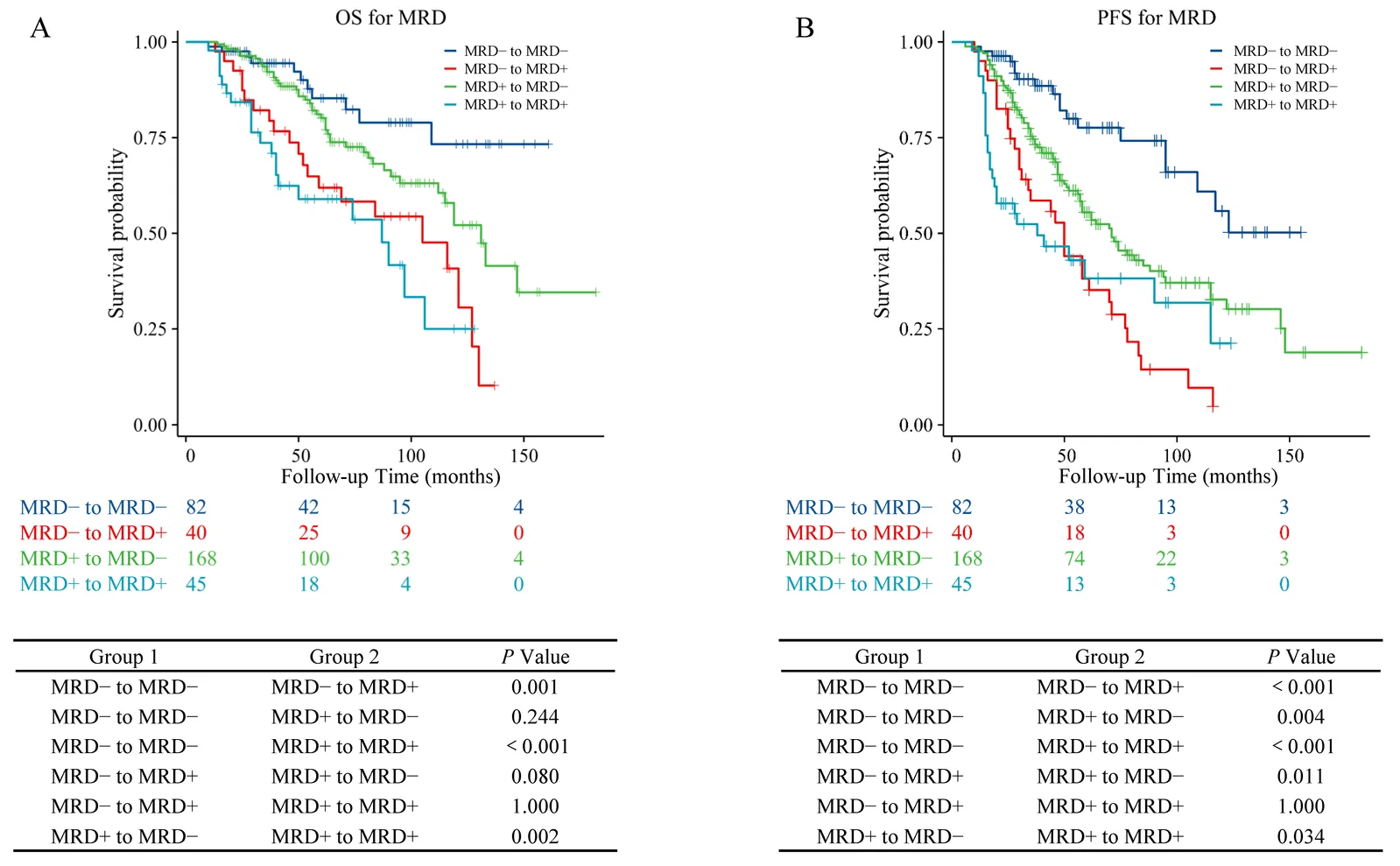
Overall survival (OS) and progression-free survival (PFS) according to minimal residual disease (MRD) dynamic patterns. Kaplan–Meier curves of OS (**A**) and PFS (**B**) are shown according to changes in MRD status between the pre-transplant and post-transplant assessments. Patients were categorized into four groups based on MRD status conversion: MRD− to MRD−, MRD− to MRD+, MRD+ to MRD−, and MRD+ to MRD+. Solid lines represent estimated survival functions. Numbers at risk at selected follow-up times are shown below each panel. Pairwise comparisons between groups were performed using the log-rank test, and *p*-values are summarized in the tables below each panel.

Figure 2A illustrates Kaplan–Meier estimates of OS according to MRD dynamic patterns. During follow-up, 99 deaths were recorded in the overall cohort. Median OS was not reached in the persistent-negative group and was 105 months in the MRD− to MRD+ group, 131 months in the MRD+ to MRD− group, and 87 months in the persistent MRD-positive group (Supplementary Table S3) (*p* < 0.001). Using the persistent MRD-negative group as the reference, patients with persistent MRD positivity had significantly worse OS (*p* < 0.001). Patients with MRD− to MRD+ conversion also showed inferior OS compared with the persistent MRD-negative group (*p* = 0.001). Although the Kaplan–Meier curves suggested a trend toward better OS in patients with persistent MRD negativity than in those with MRD+ to MRD− conversion, this difference did not reach statistical significance (*p* = 0.244). Among the non-persistent MRD-negative groups, patients with persistent MRD positivity had significantly worse OS than those with MRD+ to MRD− conversion (*p* = 0.002). OS appeared similar between the MRD− to MRD+ and persistent MRD-positive groups (*p* = 1.000). In addition, OS tended to be poorer in patients with MRD− to MRD+ conversion than in those with MRD+ to MRD− conversion, with a borderline difference (*p* = 0.080).

**Figure 3:**
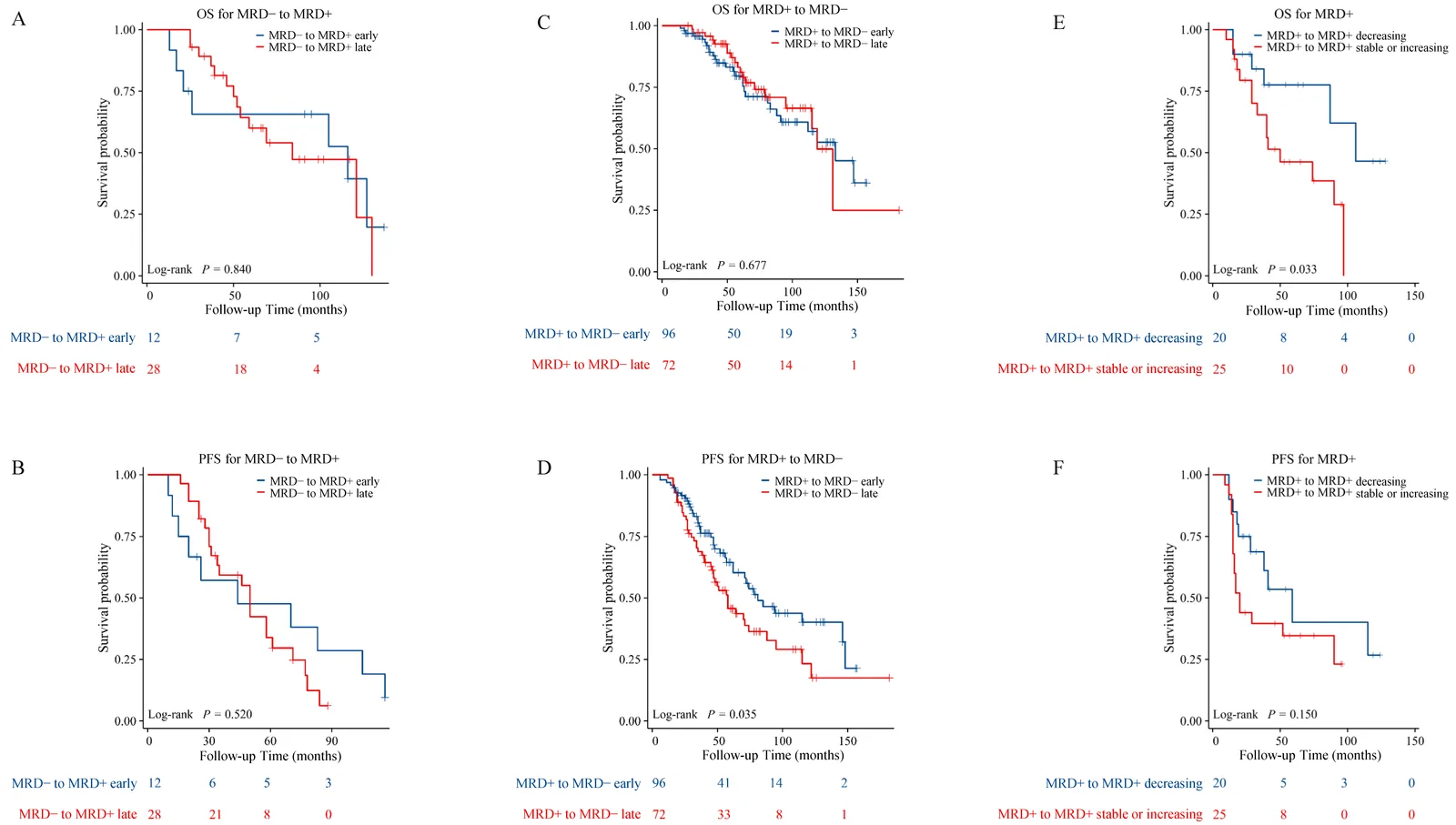
Overall survival (OS) and progression-free survival (PFS) according to minimal residual disease (MRD) status conversion before and after ASCT. Kaplan–Meier survival curves are shown for patients stratified by MRD status changes between the pre-transplant and post-transplant assessments. (**A**) OS in patients converting from MRD-negative to MRD-positive (MRD− to MRD+) status, stratified by early versus late post-transplant MRD conversion. (**B**) PFS in patients converting from MRD− to MRD+, stratified by early versus late conversion. (**C**) OS in patients converting from MRD-positive to MRD-negative (MRD+ to MRD−) status, stratified by early versus late post-transplant conversion. (**D**) PFS in patients converting from MRD+ to MRD−, stratified by early versus late conversion. (**E**) OS in patients with persistent MRD positivity, comparing those with decreasing MRD burden versus stable or increasing MRD burden over time. (**F**) PFS in patients with persistent MRD positivity, comparing decreasing MRD burden versus stable or increasing MRD burden over time.

Median OS was 116 months in the early MRD− to MRD+ group and 84 months in the late MRD− to MRD+ group (Figure 3A; *p* = 0.840). Among patients with MRD+ to MRD− conversion, OS did not differ significantly between early and late converters, with median OS of 133 months in the early group and 119 months in the late group (Figure 3C; *p* = 0.677). In contrast, among patients with persistent MRD positivity, those with decreasing MRD burden had a longer median OS than those with stable or increasing MRD burden (106 months vs. 50 months; Figure 3E; *p* = 0.033).

### 3.3. Progression-Free Survival According to MRD Dynamic Patterns

Figure 2B shows Kaplan–Meier estimates of PFS according to MRD dynamic patterns. During follow-up, 161 progression events were recorded in the overall cohort. Median PFS was not reached in the persistent MRD-negative group and was 50 months in the MRD− to MRD+ group, 71 months in the MRD+ to MRD− group, and 38 months in the persistent MRD-positive group (Supplementary Table S3) (*p* < 0.001). Using the persistent MRD-negative group as the reference, patients with MRD− to MRD+ conversion had significantly shorter PFS (*p* < 0.001). Patients with MRD+ to MRD− conversion also showed significantly inferior PFS compared with the persistent MRD-negative group (*p* = 0.004). Persistent MRD positivity was likewise associated with significantly worse PFS than persistent MRD negativity (*p* < 0.001). Among the non-persistent negative groups, PFS was significantly longer in patients with MRD+ to MRD− conversion than in those with MRD− to MRD+ conversion (*p* = 0.011) and those with persistent MRD positivity (*p* = 0.034). PFS appeared similar between the MRD− to MRD+ and persistent MRD-positive groups (*p* = 1.000).

Among patients with MRD− to MRD+ conversion, Kaplan–Meier showed no significant difference in PFS between early and late conversion groups (Figure 3B; *p* = 0.520). In contrast, among patients with MRD+ to MRD− conversion, PFS was significantly longer in early converters than in late converters, with a median PFS of 81 months versus 58 months (Figure 3D; *p* = 0.035). Among patients with persistent MRD positivity, no statistically significant difference in PFS was observed between those with decreasing MRD burden and those with stable or increasing MRD burden, although a numerical separation of the Kaplan–Meier curves was noted. Median PFS was 59 months in patients with decreasing MRD burden and 20 months in those with stable or increasing MRD burden (Figure 3F; *p* = 0.150).

The 6-month landmark sensitivity analysis showed significant differences in OS and PFS among the four MRD dynamic groups (*p* = 0.004 and *p* < 0.001, respectively), suggesting that the observed association was not solely attributable to immortal time bias (Supplementary Figure S1).

### 3.4. Univariate and Multivariable Analyses of OS According to the MRD Dynamic Pattern

In univariate analysis, factors significantly associated with OS included R-ISS staging, hemoglobin, serum LDH levels, HRCAs, cytogenetic abnormality, and maintenance treatment (Table 4). After adjustment for these prognostically relevant covariates, MRD dynamic status remained an independent predictor of OS. Compared with patients who remained MRD-negative, those who were persistently MRD-positive were associated with the highest risk of death (HR, 4.762; 95%CI, 2.151–10.546; *p* < 0.001), followed by conversion from MRD− to MRD+ (HR, 3.925; 95% CI, 1.791–8.598; *p* < 0.001), whereas conversion from MRD+ to MRD− showed a numerically increased but borderline-significant risk (HR, 1.973; 95%CI, 0.963–4.046; *p* = 0.063).

To assess the robustness of these findings, additional analyses were performed using alternative MRD categories as reference groups (Supplementary Table S4). When persistent MRD positivity was used as the reference, patients who converted from MRD-negative to MRD-positive (MRD− to MRD+) exhibited a comparable OS (HR, 0.824; 95% CI, 0.428–1.586; *p* = 0.562).

In multivariable Cox regression analyses adjusted for predefined clinical covariates (Table 5), the timing of MRD− to MRD+ conversion was not independently associated with OS. Late MRD− to MRD+ conversion showed no significant difference in OS compared with early conversion (HR, 0.962; 95% CI, 0.346–2.676; *p* = 0.942). Similarly, among patients with MRD+ to MRD− conversion, OS did not differ significantly between late and early converters after multivariable adjustment (HR, 0.968; 95% CI, 0.499–1.876; *p* = 0.923). In addition, compared with patients with decreasing MRD burden, those with stable or increasing MRD burden showed a higher risk of death after adjustment, with a borderline level of statistical significance (HR, 4.518; 95% CI, 0.956–21.340; *p* = 0.057).

### 3.5. Univariate and Multivariable Analyses of PFS According to MRD Dynamic Patterns

In univariate analysis for PFS, age, R-ISS staging, HRCAs, cytogenetic abnormality, maintenance treatment, and response at ASCT were significantly associated with PFS (Table 6). After multivariable adjustment, MRD dynamic status remained independently associated with PFS. Compared with patients who remained MRD-negative, both MRD− to MRD+ conversion (HR, 4.432; 95% CI, 2.452–8.012; *p* < 0.001) and persistent MRD positivity (HR, 4.402; 95% CI, 2.293–8.454; *p* < 0.001) were associated with markedly increased risks of disease progression, whereas MRD+ to MRD− conversion was associated with a more moderate increase in risk (HR, 2.319; 95% CI, 1.377–3.906; *p* = 0.002).

To further evaluate the robustness of these associations, additional analyses were conducted using alternative MRD categories as reference groups (Supplementary Table S4). When persistent MRD positivity was used as the reference, patients who converted from MRD− to MRD+ demonstrated a comparable risk of progression (HR, 1.007; 95% CI, 0.574–1.765; *p* = 0.981), indicating similar PFS outcomes between these two unfavorable MRD trajectories.

For PFS, late MRD− to MRD+ conversion was not independently associated with PFS compared with early conversion (HR, 1.070; 95% CI, 0.415–2.758; *p* = 0.888). However, among patients with MRD+ to MRD− conversion, late conversion was independently associated with inferior PFS compared with early conversion (HR, 1.857; 95% CI, 1.136–3.034; *p* = 0.014). Patients with stable or increasing MRD burden had a significantly higher risk of disease progression than those with decreasing MRD burden after multivariable adjustment (HR, 4.397; 95% CI, 1.190–16.240; *p* = 0.026).

In addition, we tested the proportional hazards assumption for the multivariable Cox models of OS and PFS according to dynamic MRD patterns. No significant time-varying effects were identified (Supplementary Table S5).

## 4. Discussion

In recent years, research has demonstrated that dynamic monitoring of MRD not only helped identify the progression trends of MM but also provided crucial insights for personalized treatment, enabling dynamic risk-adapted management based on MRD [12,22]. This shift allowed treatment strategies to evolve from traditional binary MRD endpoints to more refined and individualized approaches [15,24]. Previous studies have demonstrated the prognostic value of longitudinal MRD assessment. Gu et al. identified distinct MRD evolution patterns associated with outcomes in transplant-eligible patients [18]. de Tute et al. and Ferrero et al. further demonstrated the prognostic relevance of serial post-ASCT MRD assessment and MRD kinetics [19,23]. Against this context, we performed a real-world longitudinal analysis of 335 transplant-eligible MM patients treated over a 15-year period, with a median follow-up of 127 months. In addition to evaluating longitudinal MRD trajectories, we specifically examined the prognostic implications of the timing of post-ASCT MRD conversion and quantitative MRD burden changes. To the best of our knowledge, this was one of the longest follow-up studies to date on MRD status in MM patients. The primary finding of this study was that persistent MRD negativity conferred the most favorable prognosis, followed by MRD+ to MRD− conversion, whereas MRD− to MRD+ conversion and persistent MRD positivity were associated with the poorest outcomes. This further illustrated that MRD trajectory, rather than static MRD status, provided biologically and clinically meaningful prognostic information.

In our study, patients with MRD− to MRD+ demonstrated survival outcomes similar to those with persistent MRD positivity. However, a previous study suggested that persistent MRD-positive patients might have worse outcomes than those who convert from MRD− to MRD+ [20]. One possible explanation for this discrepancy lies in differences in response depth within the MRD-positive subgroup. In our study, a relatively high proportion of persistent MRD-positive patients achieved CR. Emerging evidence indicated that, even among MRD-positive patients, those achieving CR had superior survival compared with those who failed to reach CR [25]. Therefore, the prognostic impact of persistent MRD positivity might be partially modulated by the depth of conventional response, which could attenuate the survival disadvantage in this subgroup and contribute to the observed pattern.

In our study, patients who converted from MRD+ to MRD− had inferior survival outcomes compared with those with persistent MRD negativity. This observation is consistent with accumulating evidence suggesting that the timing and durability of MRD negativity are critical determinants of prognosis in multiple myeloma [13]. Taken together, these findings suggest that earlier achievement and longer persistence of MRD negativity reflect deeper disease eradication and a more favorable tumor biology, whereas delayed MRD clearance might indicate residual clonal populations that remain sensitive to therapy but are not fully eliminated.

In subgroup analysis, early conversion from MRD+ to MRD− was associated with superior PFS compared with delayed clearance, suggesting that rapid MRD eradication might reflect greater treatment sensitivity and more durable disease control. In the TOURMALINE-MM3 and -MM4 trials, MRD conversion from positive to negative within the first 14 months after ASCT was associated with significantly improved PFS compared with persistent MRD positivity, whereas this difference was no longer observed at 28 months [14]. These findings are consistent with our results, suggesting that earlier MRD clearance was associated with more favorable clinical outcomes. Similar conclusions have also been reported in studies by Lucia Y. Chen [13] and Joaquin Martinez-Lopez [4], which demonstrated that early achievement of MRD negativity is associated with improved survival outcomes in multiple myeloma. Furthermore, some studies have suggested that the prognosis associated with MRD conversion to negativity might be particularly pronounced in patients with R-ISS stage III disease [26], highlighting the potential importance of early MRD clearance in biologically high-risk populations.

In contrast, MRD conversion to positivity was associated with inferior outcomes irrespective of timing in our cohort. The similarly poor outcomes observed in patients with MRD-negative to MRD-positive conversion and those with persistent MRD positivity may reflect several biological and methodological factors. Re-emergence of MRD after an initially negative assessment may indicate clonal evolution or expansion of treatment-resistant subclones under therapeutic pressure, representing a biologically aggressive disease state similar to persistent MRD positivity. In addition, spatial heterogeneity of bone marrow involvement and sampling variability may lead to transient false-negative MRD results, particularly when residual disease is patchy or located outside the sampled site. Finally, MRD reappearance may precede biochemical or clinical relapse by several months, suggesting that conversion to MRD positivity identifies an early phase of renewed clonal expansion [20]. These mechanisms may partly explain why patients with MRD-negative to MRD-positive conversion had outcomes comparable to those with persistent MRD positivity. Notably, prior studies have reported that early MRD re-emergence (<10 years from diagnosis) is associated with significantly shorter PFS and OS, whereas late conversion (>10 years) might not compromise overall survival compared with persistent MRD negativity [20]. The discrepancy between these observations and our findings might be explained by differences in landmark definitions, and the operationalization of “early” versus “late” conversion could substantially influence risk stratification. Moreover, when adopting a 10-year landmark from diagnosis, non-myeloma-related mortality becomes increasingly relevant, including age-related frailty, comorbid conditions, and secondary malignancies, which might substantially influence overall survival and even PFS estimates. Long-term data suggested that MRD conversion might occur even after prolonged remission. Notably, the median interval between MRD re-emergence and clinical relapse was approximately one year. In high-risk patients, the interval between MRD conversion to positivity and overt relapse might be substantially shorter [20], indicating the existence of a potentially actionable preclinical window.

Quantitative MRD kinetics refine prognostication beyond categorical MRD status. Among persistently MRD-positive patients, stable or increasing MRD burden identified a biologically aggressive subset. These findings supported the concept that MRD kinetics capture clonal fitness and therapeutic pressure. Decreasing MRD burden might reflect continued responsiveness to maintenance therapy and potentially precede eventual conversion to MRD negativity [27,28]. Conversely, rising MRD burden likely represents early clonal re-expansion before overt biochemical relapse. Our finding is consistent with the results of a study by Ferrero et al., which found that patients with a pattern of MRD that was stable or increasing over time had a significantly shorter OS than patients with a pattern of MRD that was decreasing [23]. Using NGS-based quantitative MRD assessment, Bal et al. demonstrated a further reduction in myeloma disease burden following ASCT after quadruplet induction therapy, highlighting the ability of serial quantitative MRD measurements to capture treatment-related changes in residual disease burden [29]. Additionally, different MRD kinetics could be more useful for comparing the efficacy of different treatment strategies and for implementing individualized therapy-monitoring strategies.

Our findings regarding the prognostic importance of persistent MRD negativity are consistent with recent studies conducted using more sensitive MRD techniques and contemporary anti-CD38-based regimens. In analyses of the MAIA and ALCYONE trials [30], daratumumab-containing regimens substantially increased the rates and durability of MRD negativity, as assessed by next-generation sequencing. MRD negativity sustained for at least 6 or 12 months was associated with superior progression-free survival, supporting the prognostic importance of the durability of MRD response rather than a single negative assessment. Similar findings have been reported in transplant-eligible patients receiving anti-CD38-based quadruplet therapy. In the MASTER trial [28], patients received daratumumab, carfilzomib, lenalidomide, and dexamethasone followed by ASCT and MRD response-adapted consolidation. Serial MRD assessments were performed using NGS, and patients who achieved MRD negativity in two consecutive treatment phases were permitted to discontinue therapy and enter MRD surveillance. These findings indicate that sustained MRD negativity provides greater prognostic information than a single MRD-negative assessment. Although MRD was assessed using conventional multiparameter flow cytometry in the present cohort and only a small proportion of patients received anti-CD38 monoclonal antibodies, our results similarly showed that persistent MRD negativity was associated with the most favorable long-term outcomes. Nevertheless, direct comparisons with contemporary trials should be interpreted cautiously because of differences in assay sensitivity, treatment intensity, MRD assessment schedules, and patient characteristics.

This study is strengthened by prolonged follow-up, comprehensive serial MRD integration, and multivariable adjustment. Nevertheless, several limitations warrant consideration. First, the study spanned a long inclusion period of 15 years, during which substantial changes occurred in induction and maintenance regimens, supportive care, transplant practice, and MRD technologies. Anti-CD38-based therapy has been shown to improve treatment response and survival outcomes in multiple myeloma [31]; however, only a small proportion of patients in our cohort received such therapy, precluding a reliable subgroup analysis. Although treatment-related factors were included in the multivariable models, residual confounding related to treatment era, treatment intensity, and evolving clinical practice cannot be completely excluded. In addition, although MRD assessments were performed in the same institutional laboratory using broadly consistent procedures, potential variability related to reagent lots, instrument performance, and assay implementation over the 15-year period cannot be completely excluded and may have introduced measurement variability. Second, next-generation flow (NGF) and next-generation sequencing (NGS), which enable MRD detection at sensitivities approaching 10^−6^, are now routinely incorporated into contemporary clinical trials. However, MRD was assessed throughout the study using conventional multiparameter flow cytometry rather than NGS or NGF. Future studies employing deeper sequencing-based approaches might further refine longitudinal kinetic modeling. A third limitation is that we acknowledge this study is a retrospective observational study. Despite adjusting for multiple factors, there remain unavoidable confounding factors. Future prospective studies should be conducted to validate the findings of this study. Fourth, although the 6-month landmark analysis yielded results consistent with the primary analyses, immortal time bias cannot be completely excluded, particularly for MRD conversions occurring beyond the landmark period. In addition, the 3-month cutoff used to distinguish early from late conversion, although clinically based and prespecified, remains somewhat arbitrary. In addition, subsequent MRD assessment intervals were not completely uniform in this real-world cohort. Therefore, the observed survival differences should be interpreted as prognostic associations rather than causal effects. Moreover, a small proportion of patients were excluded because of sporadic missing MRD data rather than early progression or mortality. Although selection bias was likely limited, it cannot be completely excluded. Fifth, several subgroup analyses involved small sample sizes and limited numbers of outcome events, resulting in wide confidence intervals and reduced statistical power. Multiple subgroup and pairwise comparisons may also increase the risk of Type I error and false-positive findings. Accordingly, these analyses are considered exploratory and should be interpreted with caution until validated in larger independent cohorts.

## 5. Conclusions

In conclusion, longitudinal MRD trajectories provided independent and incremental prognostic information beyond static MRD assessment in patients with MM. Persistent MRD negativity conferred the best prognosis, followed by MRD+ to MRD− conversion, while persistent MRD positivity and MRD− to MRD+ conversion were associated with the worst outcomes. Furthermore, for patients who transitioned from MRD+ to MRD−, earlier conversion to MRD negativity correlated with better PFS. These findings suggest that longitudinal MRD trajectories might provide additional prognostic information beyond binary MRD endpoints and help refine risk stratification.

## Figures and Tables

**Table 1:** Baseline characteristics of patients according to MRD dynamic patterns.

Category	Total	MRD− to MRD−	MRD− to MRD+	MRD+ to MRD−	MRD+ to MRD+	*p*-Value
	Number (%)	Number (%)	Number (%)	Number (%)	Number (%)	
Gender						
Male	191 (57.0)	42 (51.2)	24 (60.0)	106 (63.1)	19 (42.2)	0.050
Female	144 (43.0)	40 (48.8)	16 (40.0)	62 (36.9)	26 (57.8)	
Age						
<55	168 (50.1)	40 (48.8)	22 (55.0)	79 (47.0)	27 (60.0)	0.417
≥55	167 (49.9)	42 (51.2)	18 (45.0)	89 (53.0)	18 (40.0)	
M-component						
IgG	166 (49.6)	37 (45.1)	22 (55.0)	79 (47.0)	28 (62.2)	0.282
IgA	73 (21.8)	24 (29.3)	9 (22.5)	36 (21.4)	4 (8.9)	
Light chain	75 (22.4)	18 (22.0)	8 (20.0)	39 (23.2)	10 (22.2)	
Other	21 (6.3)	3 (3.7)	1 (2.5)	14 (8.3)	3 (6.7)	
ISS staging						
I	126 (37.7)	39 (47.6)	8 (20.0)	62 (36.9)	17 (37.8)	0.052
II	115 (34.4)	26 (31.7)	21 (52.5)	56 (33.3)	12 (26.7)	
III	93 (27.8)	17 (20.7)	11 (27.5)	50 (29.8)	15 (33.3)	
Unknown	1 (0.0)	0 (0.0)	0 (0.0)	0 (0.0)	1(2.2)	
RISS staging						
I	98 (29.4)	32 (39.0)	7 (17.5)	49 (29.2)	10 (22.2)	0.200
II	198 (59.5)	43 (52.4)	26 (65.0)	101 (60.1)	28 (62.2)	
III	37 (11.1)	7 (8.5)	7 (17.5)	17 (10.2)	6 (13.3)	
Unknown	1 (0.0)	0 (0.0)	0 (0.0)	1(0.6)	1(2.2)	
Hemoglobin(g/dL)						
≥110	120 (35.9)	33 (40.2)	13 (32.5)	62 (36.9)	12 (26.7)	0.449
<110	214 (64.1)	49 (59.8)	27 (67.5)	105 (62.5)	33 (73.3)	
Unknown	1 (0.0)	0 (0.0)	0 (0.0)	1(0.6)	0 (0.0)	
Serum LDH levels						
≤240	270 (80.6%)	73 (89.0%)	30 (75.0%)	133 (79.2%)	34 (75.6%)	0.123
>240	60 (17.9%)	8 (9.8%)	10 (25.0%)	32 (19.0%)	10 (22.2%)	
Unknown	5 (1.5%)	1 (1.2%)	0 (0.0%)	3 (1.8%)	1 (2.2%)	
HRCAs						
No	264 (78.8%)	67 (81.7%)	29 (72.5%)	135 (80.4%)	33 (73.3%)	0.321
Yes	67 (20.0%)	13 (15.9%)	11 (27.5%)	31 (18.5%)	12 (26.7%)	
Unknown	4 (1.2%)	2 (2.4%)	0 (0.0%)	2 (1.2%)	0 (0.0%)	
Cytogenetic abnormality						
0	115 (34.3%)	32 (39.0%)	14 (35.0%)	59 (35.1%)	10 (22.2%)	0.447
1	103 (30.7%)	22 (26.8%)	12 (30.0%)	55 (32.7%)	14 (31.1%)	
≥2	113 (33.7%)	26 (31.7%)	14 (35.0%)	52 (31.0%)	21 (46.7%)	
Unknown	4 (1.2%)	2 (2.4%)	0 (0.0%)	2 (1.2%)	0 (0.0%)	
Induction treatment						
PI-based	244 (72.8)	65 (79.3)	28 (70.0)	122 (72.6)	29 (64.4)	0.083
IMiD-based	18 (5.4)	2 (2.4)	5 (12.5)	6 (3.6)	5 (11.1)	
PI + IMiD-based	47 (14.0)	9 (11.0)	2 (5.0)	28 (16.7)	8 (17.8)	
Other	26 (7.8)	6 (7.3)	5 (12.5)	12 (7.1)	3 (6.7)	
Maintenance treatment						
PI-based	47 (14.0)	10 (12.2)	7 (17.5)	24 (14.3)	6 (13.3)	0.779
IMiD-based	214 (63.9)	49 (59.8)	27 (67.5)	109 (64.9)	29 (64.4)	
PI + IMiD-based	25 (7.5)	8 (9.8)	2 (5.0)	14 (8.3)	1 (2.2)	
Other	43 (12.8)	14 (17.1)	4 (10.0)	17 (10.1)	8 (17.8)	
No maintenance	6 (1.8)	1 (1.2)	0 (0.0)	4 (2.4)	1 (2.2)	
Anti-CD38-based therapy						
No	293 (87.5%)	68 (82.9%)	36 (90.0%)	149 (88.7%)	40 (88.9%)	0.544
Yes	42 (12.5%)	14 (17.1%)	4 (10.0%)	19 (11.3%)	5 (11.1%)	
Response at ASCT						
≥CR	141 (42.1)	50 (61.0)	19 (47.5)	67 (39.9)	5 (11.1)	<0.001
<CR	194 (57.9)	32 (39.0)	21 (52.5)	101 (60.1)	40 (88.9)	
Complications						
0	118 (35.2)	23 (28.0)	15 (37.5)	63 (37.5)	17 (37.8)	0.654
1	141 (42.1)	38 (46.3)	17 (42.5)	65 (38.7)	21 (46.7)	
≥2	76 (22.7)	21 (25.6)	8 (20.0)	40 (23.8)	7 (15.6)	

Abbreviations: HRCAs, high-risk cytogenetic abnormalities, including del 17p; t(4;14), t(14;16) or t(14;20); ASCT, autologous stem cell transplantation; LDH, lactate dehydrogenase; ISS, International Staging System; R-ISS, Revised International Staging System; MRD, minimal residual disease; ≥CR, patient achieves complete response (CR) or better (sCR); <CR, less than complete response; PI, proteasome inhibitor; IMiD, immunomodulatory drug. Unknown values were excluded from the statistical comparison.

**Table 2:** Baseline characteristics stratified by early (≤3 months post-ASCT) and late (>3 months post-ASCT) MRD status among patients with MRD conversion.

Category	MRD− to MRD+			*p*-Value	MRD+ to MRD−			*p*-Value
	Total	Early	Late		Total	Early	Late	
	Number (%)	Number (%)	Number (%)		Number (%)	Number (%)	Number (%)	
Gender								
Male	24 (60.0)	7 (58.3)	17 (60.7)	0.888	106 (63.1)	57 (59.4)	49 (68.1)	0.249
Female	16 (40.0)	5 (41.7)	11 (39.3)		62 (36.9)	39 (40.6)	23 (31.9)	
Age								
<55	22 (55.0)	7 (58.3)	15 (53.6)	0.781	79 (47.0)	47 (49.0)	32 (44.4)	0.562
≥55	18 (45.0)	5 (41.7)	13 (46.4)		89 (53.0)	49 (51.0)	40 (55.6)	
M-component								
IgG	22 (55.0)	8 (66.7)	14 (50.0)	0.751	79 (47.0)	47 (49.0)	32 (44.4)	0.882
IgA	9 (22.5)	2 (16.7)	7 (25.0)		36 (21.4)	21 (21.9)	15 (20.8)	
Light chain	8 (20.0)	2 (16.7)	6 (21.4)		39 (23.2)	21 (21.9)	18 (25.0)	
Other	1 (2.5)	0 (0.0)	1 (3.6)		14 (8.3)	7 (7.3)	7 (9.7)	
ISS staging								
I	8 (20.0)	3 (25.0)	5 (17.9)	0.589	62 (36.9)	34 (35.4)	28 (38.9)	0.500
II	21 (52.5)	7 (58.3)	14 (50.0)		56 (33.3)	30 (31.3)	26 (36.1)	
III	11 (27.5)	2 (16.7)	9 (32.1)		50 (29.8)	32 (33.3)	18 (25.0)	
RISS staging								
I	7 (17.5)	3 (25.0)	4 (14.3)	0.714	49 (29.2%)	26 (27.1%)	23 (31.9%)	0.219
II	26 (65.0)	7 (58.3)	19 (67.9)		101 (60.1%)	56 (58.3%)	45 (62.5%)	
III	7 (17.5)	2 (16.7)	5 (17.9)		17 (10.1%)	13 (13.5%)	4 (5.6%)	
Unknown	0 (0.0%)	0 (0.0%)	0 (0.0%)		1 (0.6%)	1 (1.0%)	0 (0.0%)	
Hemoglobin(g/dL)								
≥110	13 (32.5)	3 (25.0)	10 (35.7)	0.507	62 (36.9%)	33 (34.4%)	29 (40.3%)	0.463
<110	27 (67.5)	9 (75.0)	18 (64.3)		105 (62.5%)	62 (64.6%)	43 (59.7%)	
Unknown	0 (0.0%)	0 (0.0%)	0 (0.0%)		1 (0.6%)	1 (1.0%)	0 (0.0%)	
Serum LDH levels								
≤240	30 (75.0)	8 (66.7)	22 (78.6)	0.426	133 (79.2%)	72 (75.0%)	61 (84.7%)	0.134
>240	10 (25.0)	4 (33.3)	6 (21.4)		32 (19.0%)	22 (22.9%)	10 (13.9%)	
Unknown	0 (0.0%)	0 (0.0%)	0 (0.0%)		3 (1.8%)	2 (2.1%)	1 (1.4%)	
HRCAs								
No	29 (72.5)	9 (75.0)	20 (71.4)	0.817	135 (80.4%)	78 (81.3%)	57 (79.2%)	0.765
Yes	11 (27.5)	3 (25.0)	8 (28.6)		31 (18.5%)	17 (17.7%)	14 (19.4%)	
Unknown	0 (0.0%)	0 (0.0%)	0 (0.0%)		2 (1.2%)	1 (1.0%)	1 (1.4%)	
Cytogenetic abnormality								
0	14 (35.0)	6 (50.0)	8 (28.6)	0.344	59 (35.1%)	32 (33.3%)	27 (37.5%)	0.311
1	12 (30.0)	2 (16.7)	10 (35.7)		55 (32.7%)	36 (37.5%)	19 (26.4%)	
≥2	14 (35.0)	4 (33.3)	10 (35.7)		52 (31.0%)	27 (28.1%)	25 (34.7%)	
Unknown	0 (0.0%)	0 (0.0%)	0 (0.0%)		2 (1.2%)	1 (1.0%)	1 (1.4%)	
Induction treatment								
PI-based	28 (70.0)	10 (83.3)	18 (64.3)	0.622	122 (72.6)	71 (74.0)	51 (70.8)	0.383
IMiD-based	5 (12.5)	1 (8.3)	4 (14.3)		6 (3.6)	3 (3.1)	3 (4.2)	
PI + IMiD-based	2 (5.0)	0 (0.0)	2 (7.1)		28 (16.7)	13 (13.5)	15 (20.8)	
Other	5 (12.5)	1 (8.3)	4 (14.3)		12 (7.1)	9 (9.4)	3 (4.2)	
Maintenance treatment								
PI-based	7 (17.5)	1 (8.3)	6 (21.4)	0.314	24 (14.3)	12 (12.5)	12 (16.7)	0.243
IMiD-based	27 (67.5)	10 (83.3)	17 (60.7)		109 (64.9)	59 (61.5)	50 (69.4)	
PI + IMiD-based	2 (5.0)	1 (8.3)	1 (3.6)		14 (8.3)	10 (10.4)	4 (5.6)	
Other	4 (10.0)	0 (0.0)	4 (14.3)		17 (10.1)	11 (11.5)	6 (8.3)	
No maintenance	0 (0.0)	0 (0.0)	0 (0.0)		4 (2.4)	4 (4.2)	0 (0.0)	
Anti-CD38-based therapy								
No	36 (90.0)	11 (91.7)	25 (89.3)	0.818	149 (88.7%)	83 (86.5%)	66 (91.7%)	0.291
Yes	4 (10.0)	1 (8.3)	3 (10.7)		19 (11.3%)	13 (13.5%)	6 (8.3%)	
Response at ASCT								
≥CR	19 (47.5)	5 (41.7)	14 (50.0)	0.629	67 (39.9)	42 (43.8)	25 (34.7)	0.237
<CR	21 (52.5)	7 (58.3)	14 (50.0)		101 (60.1)	54 (56.3)	47 (65.3)	
Complications								
0	15 (37.5)	6 (50.0)	9 (32.1)	0.089	63 (37.5)	33 (34.4)	30 (41.7)	0.577
1	17 (42.5)	4 (33.3)	13 (46.4)		65 (38.7)	38 (39.6)	27 (37.5)	
≥2	8 (20.0)	2 (16.7)	6 (21.4)		40 (23.8)	25 (26.0)	15 (20.8)	

Abbreviations: HRCAs, high-risk cytogenetic abnormalities, including del 17p; t(4;14), t(14;16) or t(14;20); ASCT, autologous stem cell transplantation; LDH, lactate dehydrogenase; ISS, International Staging System; MRD, minimal residual disease; ≥CR, patient achieves complete response (CR) or better (sCR); nCR, not complete response; PI: proteasome inhibitor; IMiD: immunomodulatory drug. Unknown values were excluded from the statistical comparison.

**Table 3:** Baseline characteristics in multiple myeloma patients with persistently positive MRD.

Category	MRD+ to MRD+			*p* Value
	Total	Decreasing	Stable or Increasing	
	Number (%)	Number (%)	Number (%)	
Gender				
Male	19 (42.2)	8 (40.0)	11 (44.0)	0.787
Female	26 (57.8)	12 (60.0)	14 (56.0)	
Age				
<55	27 (60.0)	9 (45.0)	18 (72.0)	0.066
≥55	18 (40.0)	11 (55.0)	7 (28.0)	
M-component				
IgG	28 (62.2)	14 (70.0)	14 (56.0)	0.164
IgA	4 (8.9)	3 (15.0)	1 (4.0)	
Light chain	10 (22.2)	3 (15.0)	7 (28.0)	
Other	3 (6.7)	0 (0.0)	3 (12.0)	
ISS staging				
I	17 (37.8)	8 (40.0)	9 (36.0)	0.115
II	12 (26.7)	8 (40.0)	4 (16.0)	
III	15 (33.3)	4 (20.0)	11 (44.0)	
Unknown	1(2.2)	0 (0.0)	1 (4.0)	
RISS staging				
I	10 (22.2)	4 (20.0)	6 (24.0)	0.238
II	28 (62.2)	15 (75.0)	13 (52.0)	
III	6 (13.3)	1 (5.0)	5 (20.0)	
Unknown	1(2.2)	0 (0.0)	1 (4.0)	
Hemoglobin(g/dL)				
≥110	12 (26.7)	5 (25.0)	7 (28.0)	0.821
<110	33 (73.3)	15 (75.0)	18 (72.0)	
Serum LDH levels				
≤240	34 (75.6%)	16 (80.0%)	18 (72.0%)	0.694
>240	10 (22.2%)	4 (20.0%)	6 (24.0%)	
Unknown	1 (2.2%)	0 (0.0%)	1 (4.0%)	
HRCAs				
No	33 (73.3)	15 (75.0)	18 (72.0)	0.821
Yes	12 (26.7)	5 (25.0)	7 (28.0)	
Cytogenetic abnormality				
0	10 (22.2)	3 (15.0)	7 (28.0)	0.575
1	14 (31.1)	7 (35.0)	7 (28.0)	
≥2	21 (46.7)	10 (50.0)	11 (44.0)	
Induction treatment				
PI-based	29 (64.4)	14 (70.0)	15 (60.0)	0.565
IMiD-based	5 (11.1)	3 (15.0)	2 (8.0)	
PI + IMiD-based	8 (17.8)	2 (10.0)	6 (24.0)	
Other	3 (6.7)	1 (5.0)	2 (8.0)	
Maintenance treatment				
PI-based	6 (13.3)	3 (15.0)	3 (12.0)	0.309
IMiD-based	29 (64.4)	10 (50.0)	19 (76.0)	
PI + IMiD-based	1 (2.2)	1 (5.0)	0 (0.0)	
Other	8 (17.8)	5 (25.0)	3 (12.0)	
No maintenance	1 (2.2)	1 (5.0)	0 (0.0)	
Anti-CD38-based therapy				
No	40 (88.9%)	16 (80.0%)	24 (96.0%)	0.090
Yes	5 (11.1%)	4 (20.0%)	1 (4.0%)	
Response at ASCT				
≥CR	5 (11.1)	2 (10.0)	3 (12.0)	0.832
<CR	40 (88.9)	18 (90.0)	22 (88.0)	
Complications				
0	17 (37.8)	7 (35.0)	10 (40.0)	0.516
1	21 (46.7)	11 (55.0)	10 (40.0)	
≥2	7 (15.6)	2 (10.0)	5 (20.0)	

Abbreviations: HRCAs, high-risk cytogenetic abnormalities, including del 17p; t(4;14), t(14;16) or t(14;20); ASCT, autologous stem cell transplantation; LDH, lactate dehydrogenase; ISS, International Staging System; R-ISS, Revised International Staging System; MRD, minimal residual disease; ≥CR, patient achieves complete response (CR) or better (sCR); <CR, less than complete response; PI, proteasome inhibitor; IMiD, immunomodulatory drug. Unknown values were excluded from the statistical comparison.

**Table 4:** Univariate and multivariate analyses of OS for enrolled patients.

Category	N	Univariate			N	Multivariate		
		HR	95% CI	*p*-Value		HR	95% CI	*p*-Value
MRD status								
MRD− to MRD−	82	1			79	1		
MRD− to MRD+	40	3.810	1.828–7.940	<0.001	40	3.925	1.791–8.598	<0.001
MRD+ to MRD−	168	2.011	1.040–3.891	0.038	162	1.973	0.963–4.046	0.063
MRD+ to MRD+	45	5.019	2.400–10.498	<0.001	43	4.762	2.151–10.546	<0.001
Gender								
Male	191	1				-		
Female	144	1.070	0.719–1.593	0.737				
Age								
<55	168	1				-		
≥55	167	1.275	0.859–1.895	0.228				
M-component								
IgG	166	1				-		
IgA	73	1.322	0.799–2.190	0.277				
Light chain	75	1.060	0.650–1.729	0.814				
Other	21	0.522	0.163–1.674	0.274				
RISS staging								
I	94	1			94	1		
II	195	1.968	1.164–3.326	0.011	195	1.681	0.874–3.233	0.119
III	35	3.936	2.023–7.658	<0.001	35	2.823	1.045–7.630	0.041
Hemoglobin (g/dL)								
≥110	120	1			118	1		
<110	214	1.674	1.075–2.605	0.023	207	1.099	0.644–1.873	0.730
Serum LDH levels(U/L)								
≤ 240	270	1			266	1		
>240	60	1.799	1.124–2.879	0.014	59	1.523	0.842–2.756	0.164
HRCAs								
No	264	1			258	1		
Yes	67	1.354	0.852–2.153	0.199	67	0.629	0.308–1.285	0.204
Cytogenetic abnormality								
0	115	1			112	1		
1	103	0.897	0.527–1.527	0.689	101	0.610	0.344–1.084	0.092
≥2	113	1.564	0.981–2.493	0.060	112	1.354	0.695–2.635	0.373
Induction treatment								
PI-based	244	1				-		
IMiD-based	18	1.311	0.600–2.868	0.497				
PI + IMiD-based	47	1.566	0.907–2.706	0.108				
Other	26	1.088	0.437–2.709	0.856				
Maintenance treatment								
PI-based	47	1			47	1		
IMiD-based	214	1.211	0.601–2.442	0.592	205	1.239	0.602–2.549	0.561
PI + IMiD-based	25	1.008	0.216–4.715	0.992	25	0.913	0.189–4.402	0.909
Other	43	1.230	0.512–2.954	0.643	42	1.658	0.651–4.226	0.289
No maintenance	6	7.189	1.930–26.778	0.003	6	5.696	1.335–24.309	0.019
Anti-CD38-based therapy								
No	293	1				-		
Yes	42	0.350	0.086–1.435	0.145				
Response at ASCT								
≥CR	141	1				-		
<CR	194	1.331	0.883–2.007	0.173				
Complications								
0	118	1				-		
1	141	0.749	0.479–1.174	0.208				
≥2	76	0.864	0.514–1.451	0.581				

Abbreviations: HRCAs, high-risk cytogenetic abnormalities, including del 17p; t(4;14), t(14;16) or t(14;20); ASCT, autologous stem cell transplantation; LDH, lactate dehydrogenase; ISS, International Staging System; R-ISS, Revised International Staging System; MRD, minimal residual disease; ≥CR, patient achieves complete response (CR) or better (sCR); <CR, less than complete response; PI, proteasome inhibitor; IMiD, immunomodulatory drug. OS adjusted for R-ISS staging, hemoglobin, serum LDH levels, HRCAs, cytogenetic abnormality, and maintenance treatment.

**Table 6:** Univariate and multivariate analyses of PFS for enrolled patients.

Category	N	Univariate			N	Multivariate		
		HR	95% CI	*p*-Value		HR	95% CI	*p*-Value
MRD status								
MRD− to MRD−	82	1			80	1		
MRD− to MRD+	40	4.434	2.503–7.854	<0.001	40	4.432	2.452–8.012	<0.001
MRD+ to MRD−	168	2.335	1.417–3.846	0.001	165	2.319	1.377–3.906	0.002
MRD+ to MRD+	45	4.313	2.392–7.777	<0.001	44	4.402	2.293–8.454	<0.001
Gender								
Male	191	1				-		
Female	144	0.882	0.643–1.210	0.437				
Age								
<55	168	1			164	1		
≥55	167	1.309	0.959–1.785	0.090	167	1.357	0.979–1.883	0.067
M-component								
IgG	166	1				-		
IgA	73	1.149	0.765–1.724	0.504				
Light chain	75	1.034	0.698–1.532	0.868				
Other	21	1.031	0.534–1.989	0.928				
RISS staging								
I	98	1			96	1		
II	198	1.423	0.981–2.065	0.063	197	1.264	0.841–1.901	0.260
III	37	2.667	1.599–4.449	<0.001	36	2.415	1.285–4.539	0.006
Hemoglobin (g/dL)								
≥110	120	1				-		
<110	214	1.314	0.946–1.827	0.104				
Serum LDH levels								
≤240	270	1				-		
>240	60	1.323	0.888–1.973	0.169				
HRCAs								
No	264	1			263	1		
Yes	67	1.399	0.971–2.016	0.071	67	0.745	0.434–1.278	0.285
Cytogenetic abnormality								
0	115	1			114	1		
1	103	0.867	0.578–1.300	0.489	103	0.658	0.428–1.011	0.056
≥2	113	1.461	1.014–2.105	0.042	113	1.158	0.699–1.919	0.569
Induction treatment								
PI-based	244	1				-		
IMiD-based	18	1.202	0.629–2.298	0.577				
PI + IMiD-based	47	1.525	0.988–2.356	0.057				
Other	26	1.009	0.510–1.994	0.980				
Maintenance treatment								
PI-based	47	1			47	1		
IMiD-based	214	0.853	0.527–1.381	0.517	210	0.849	0.510–1.415	0.530
PI + IMiD-based	25	1.319	0.577–3.016	0.512	25	1.234	0.518–2.939	0.635
Other	43	1.126	0.602–2.108	0.710	42	1.487	0.768–2.879	0.239
No maintenance	6	4.609	1.344–15.806	0.015	6	2.782	0.727–10.640	0.135
Anti-CD38-based therapy								
No	293	1				-		
Yes	42	0.791	0.399–1.565	0.500				
Response at ASCT								
≥CR	141	1			138	1		
<CR	194	1.458	1.054–2.017	0.023	192	1.113	0.773–1.601	0.566
Complications								
0	118	1				-		
1	141	1.024	0.721–1.453	0.896				
≥2	76	0.893	0.584–1.366	0.601				

Abbreviations: HRCAs, high-risk cytogenetic abnormalities, including del 17p; t(4;14), t(14;16) or t(14;20); ASCT, autologous stem cell transplantation; LDH, lactate dehydrogenase; ISS, International Staging System; R-ISS, Revised International Staging System; MRD, minimal residual disease; ≥CR, patient achieves complete response (CR) or better (sCR); <CR, less than complete response; PI, proteasome inhibitor; IMiD, immunomodulatory drug. PFS adjusted for age, R-ISS staging, HRCAs, cytogenetic abnormality, maintenance treatment, and response at ASCT.

**Table 5:** Univariate and multivariate analyses of OS and PFS according to MRD dynamic subgroups.

Category	N	Univariate			N	Multivariate		
		HR	95% CI	*p*-Value		HR	95% CI	*p*-Value
OS								
MRD− to MRD+								
Group								
MRD− to MRD+ early	12	1			12	1		
MRD− to MRD+ late	28	1.103	0.427–2.846	0.840	28	0.962	0.346–2.676	0.942
MRD+ to MRD−								
Group								
MRD+ to MRD− early	96	1			92	1		
MRD+ to MRD− late	72	0.882	0.486–1.600	0.679	70	0.968	0.499–1.876	0.923
MRD+ to MRD+								
Group								
MRD+ to MRD+ decreasing	20	1			20	1		
MRD+ to MRD+ stable or increasing	25	2.891	1.033–8.090	0.043	23	4.518	0.956–21.340	0.057
PFS								
MRD− to MRD+								
Group								
MRD− to MRD+ early	12	1			12	1		
MRD− to MRD+ late	28	1.311	0.568–3.026	0.526	28	1.07	0.415–2.758	0.888
MRD+ to MRD−								
Group								
MRD+ to MRD− early	96	1			94	1		
MRD+ to MRD− late	72	1.582	1.026–2.441	0.038	71	1.857	1.136–3.034	0.014
MRD+ to MRD+								
Group								
MRD+ to MRD+ decreasing	20	1			20	1		
MRD+ to MRD− stable or increasing	25	1.781	0.792–4.007	0.163	24	4.397	1.190–16.240	0.026

OS adjusted for R-ISS staging, hemoglobin, serum LDH levels, HRCAs, cytogenetic abnormality, and maintenance treatment; PFS adjusted for age, R-ISS staging, HRCAs, cytogenetic abnormality, maintenance treatment, and response at ASCT.

## Data Availability

The original contributions presented in this study are included in the article and its Supplementary Materials. Further inquiries can be directed to the corresponding author.

## Supplementary Materials

The following supporting information can be downloaded at: https://www.mdpi.com/article/10.3390/cancers18182958/s1, Figure S1: Six-month landmark analysis of overall survival (OS) and progression-free survival (PFS) according to dynamic MRD patterns. (A) OS stratified by four dynamic MRD patterns: MRD− to MRD−, MRD− to MRD+, MRD+ to MRD−, and MRD+ to MRD+. (B) PFS stratified by the same dynamic MRD patterns. Numbers at risk at selected time points are displayed below each panel. Overall differences among groups were assessed using the log-rank test; Table S1: Individual-level longitudinal MRD status in the 335 included patients; Table S2: Summary table of MRD assessment frequency; Table S3: Median Overall Survival (OS) and Progression-Free Survival (PFS) according to different MRD dynamic patterns; Table S4: Univariate and multivariate analyses of OS and PFS using different MRD statuses as reference; Table S5: Proportional hazards assumption testing for MRD dynamic groups in the Cox regression model.
