# Supplementary material for: The Prognostic Significance of Dynamic Monitoring of Minimal Residual Disease Status in Patients with Multiple Myeloma: A Real-World Study
Source: Cancers (Basel). 2026 Sep 13;18(18):2958. doi: 10.3390/cancers18182958 (PMC13604359; doi:10.3390/cancers18182958)
Supplement: Supplementary file 1 [file cancers-18-02958-s001.zip › cancers-4497866-supplementary.pdf]

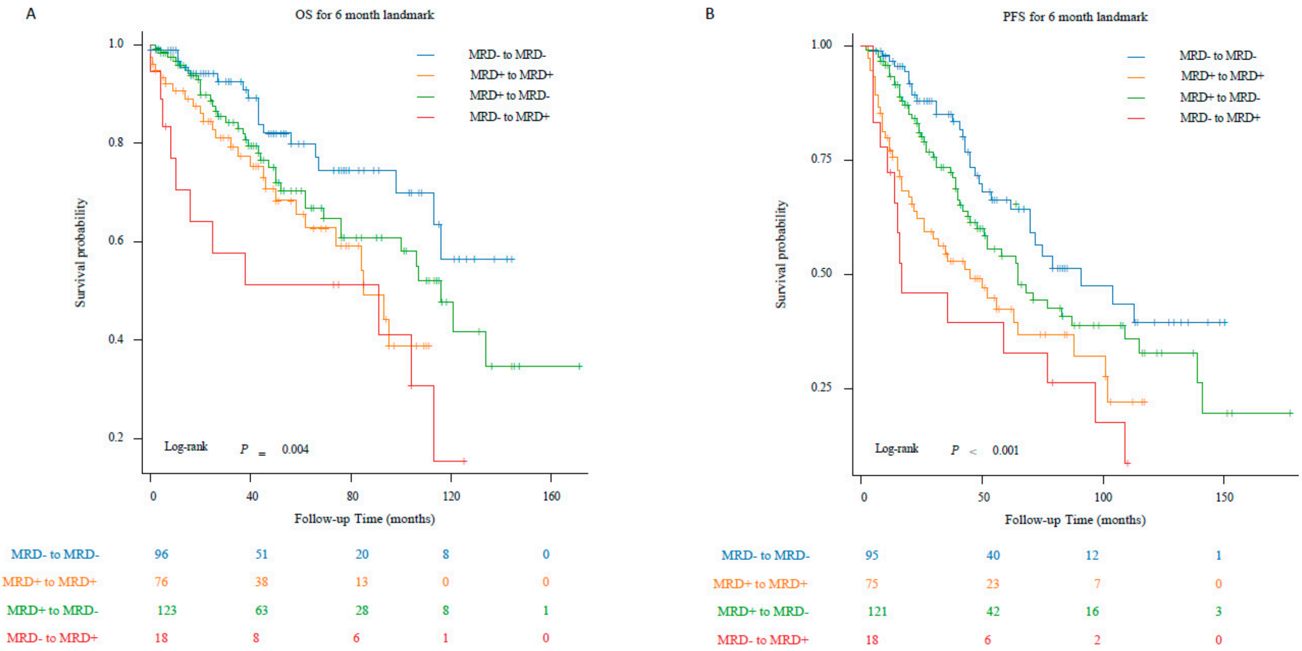

Figure S1. Six-month landmark analysis of overall survival (OS) and progression-free survival (PFS) according to dynamic MRD patterns. (A) OS stratified by four dynamic MRD patterns: MRD− to MRD−, MRD− to MRD+, MRD+ to MRD−, and MRD+ to MRD+. (B) PFS stratified by the same dynamic MRD patterns. Numbers at risk at selected time points are displayed below each panel. Overall differences among groups were assessed using the log-rank test.

Table S1. Individual-level longitudinal MRD status in the 335 included patients.

ID	MRD model	MRD-pre	MRD3m	MRD6m	MRD9m	MRD12m	MRD18m	MRD24m	MRD30m	MRD36m	Other
001	MRD+ to MRD-	positive	positive	positive	negative	positive	positive	positive	NA	NA	
002	MRD+ to MRD-	positive	negative	negative	negative	negative	negative	negative	NA	NA	
003	MRD+ to MRD+	positive	positive	positive	positive	positive	positive	positive	positive	NA	
004	MRD- to MRD-	negative	negative	NA	negative	NA	negative	negative	NA	NA	
005	MRD- to MRD+	negative	negative	negative	negative	negative	negative	positive	NA	NA	
006	MRD+ to MRD-	positive	negative	NA	NA	negative	negative	NA	NA	NA	
007	MRD+ to MRD-	positive	positive	positive	positive	negative	negative	negative	negative	NA	
008	MRD- to MRD-	negative	negative	negative	negative	negative	negative	NA	NA	NA	
009	MRD+ to MRD-	positive	negative	NA	NA	positive	positive	positive	positive	NA	
010	MRD+ to MRD-	positive	positive	positive	positive	negative	negative	negative	NA	NA	
011	MRD+ to MRD-	positive	negative	negative	NA	NA	NA	NA	NA	NA	

012	MRD- to MRD-	negative	negative	negative	negative	negative	negative	negative	negative	negative	
013	MRD+ to MRD+	positive	positive	NA	NA	NA	NA	NA	NA	NA	
014	MRD+ to MRD+	positive	positive	positive	positive	NA	NA	NA	NA	NA	
015	MRD- to MRD-	negative	negative	negative	negative	negative	negative	negative	negative	negative	
016	MRD+ to MRD-	positive	positive	positive	NA	negative	positive	negative	negative	negative	
017	MRD+ to MRD-	positive	negative	negative	negative	positive	NA	NA	NA	NA	
018	MRD+ to MRD-	positive	negative	negative	negative	negative	negative	negative	negative	negative	
019	MRD+ to MRD-	positive	negative	negative	negative	negative	negative	NA	negative	NA	
020	MRD+ to MRD-	positive	positive	negative	NA	positive	positive	negative	NA	NA	
021	MRD+ to MRD-	positive	positive	positive	positive	negative	positive	NA	NA	NA	
022	MRD+ to MRD-	positive	negative	NA	negative	negative	negative	negative	NA	NA	
023	MRD+ to MRD-	positive	positive	positive	negative	negative	negative	negative	negative	negative	
024	MRD+ to MRD-	positive	positive	negative	NA	negative	positive	positive	positive	positive	
025	MRD- to MRD-	negative	negative	negative	negative	negative	negative	negative	negative	NA	
026	MRD+ to MRD-	positive	positive	negative	negative	negative	positive	positive	positive	negative	38m positive
027	MRD- to MRD-	negative	negative	NA	NA	NA	NA	NA	NA	NA	
028	MRD+ to MRD-	positive	positive	NA	NA	negative	NA	NA	NA	NA	
029	MRD- to MRD-	negative	negative	negative	negative	negative	negative	negative	negative	negative	
030	MRD+ to MRD-	positive	negative	NA	negative	positive	negative	negative	negative	negative	
031	MRD+ to MRD-	positive	negative	negative	negative	negative	negative	negative	negative	positive	39m positive
032	MRD+ to MRD-	positive	positive	negative	negative	negative	negative	negative	negative	negative	
033	MRD+ to MRD+	positive	positive	NA	positive	NA	NA	NA	NA	NA	
034	MRD+ to MRD-	positive	negative	negative	negative	NA	NA	NA	NA	NA	
035	MRD+ to MRD-	positive	negative	negative	negative	negative	negative	negative	negative	negative	
036	MRD- to MRD-	negative	negative	NA	negative	NA	NA	NA	NA	NA	
037	MRD+ to MRD+	positive	positive	NA	NA	positive	NA	NA	NA	NA	

038	MRD+ to MRD-	positive	negative	NA	negative	NA	positive	NA	negative	negative	48m negative
039	MRD+ to MRD-	positive	negative	NA	negative	NA	negative	negative	NA	NA	40m positive
040	MRD+ to MRD+	positive	positive	positive	positive	positive	positive	positive	positive	NA	
041	MRD+ to MRD-	positive	negative	negative	negative	negative	negative	negative	NA	negative	45m negative
042	MRD+ to MRD-	positive	negative	NA	negative	negative	negative	negative	negative	negative	45m negative
043	MRD- to MRD-	negative	negative	negative	NA	NA	NA	NA	NA	NA	
044	MRD+ to MRD-	positive	negative	positive	positive	positive	negative	negative	negative	negative	48m negative
045	MRD+ to MRD-	positive	positive	NA	positive	negative	negative	negative	negative	NA	
046	MRD- to MRD-	negative	negative	negative	negative	NA	negative	negative	negative	negative	
047	MRD- to MRD+	negative	negative	negative	negative	negative	negative	NA	NA	negative	48m positive
048	MRD+ to MRD-	positive	positive	NA	negative	negative	positive	positive	NA	NA	
049	MRD+ to MRD-	positive	positive	positive	negative	negative	negative	negative	NA	negative	
050	MRD+ to MRD-	positive	positive	negative	positive	positive	positive	positive	positive	positive	48m positive
051	MRD- to MRD+	negative	negative	NA	NA	NA	negative	negative	NA	positive	48m negative
052	MRD+ to MRD-	positive	positive	NA	positive	positive	negative	positive	NA	positive	
053	MRD+ to MRD-	positive	positive	negative	NA	negative	NA	negative	NA	NA	
054	MRD+ to MRD+	positive	positive	NA	positive	positive	positive	NA	positive	positive	50m positive
055	MRD+ to MRD-	positive	positive	NA	negative	negative	negative	positive	NA	positive	42m positive
056	MRD+ to MRD-	positive	positive	NA	negative	negative	negative	NA	NA	NA	
057	MRD+ to MRD+	positive	positive	NA	NA	NA	NA	NA	NA	NA	
058	MRD+ to MRD-	positive	positive	positive	positive	negative	NA	negative	negative	negative	47m negative
059	MRD- to MRD+	negative	negative	negative	negative	negative	positive	positive	NA	NA	
060	MRD+ to MRD-	positive	negative	negative	negative	negative	negative	negative	negative	negative	50m negative
061	MRD+ to MRD-	positive	negative	positive	negative	negative	positive	negative	NA	positive	53m positive
062	MRD- to MRD+	negative	negative	negative	negative	negative	negative	negative	negative	positive	42m positive
063	MRD+ to MRD-	positive	positive	negative	negative	negative	negative	negative	negative	positive	48m negative

064	MRD+ to MRD-	positive	negative	NA	NA	NA	negative	NA	NA	NA	
065	MRD- to MRD-	negative	negative	negative	negative	negative	negative	negative	negative	negative	48m negative
066	MRD- to MRD+	negative	negative	negative	negative	negative	negative	negative	negative	positive	44m positive
067	MRD- to MRD-	negative	negative	negative	negative	negative	negative	NA	negative	NA	
068	MRD+ to MRD-	positive	positive	negative	negative	negative	negative	NA	NA	NA	
069	MRD+ to MRD-	positive	positive	positive	positive	positive	NA	negative	NA	NA	
070	MRD- to MRD-		negative	NA	negative	negative	negative	negative	NA	negative	46m negative
071	MRD+ to MRD-	positive	negative	negative	NA	negative	NA	NA	NA	NA	51m positive
072	MRD- to MRD-	negative	negative	negative	NA	negative	negative	negative	negative	negative	
073	MRD+ to MRD-	positive	positive	negative	negative	negative	negative	negative	negative	positive	42m negative
074	MRD+ to MRD+	positive	positive	positive	positive	positive	positive	positive	positive	positive	50m positive
075	MRD+ to MRD-	positive	negative	negative	negative	negative	NA	NA	NA	NA	
076	MRD+ to MRD-	positive	negative	negative	negative	positive	positive	positive	positive	positive	45m positive
077	MRD+ to MRD-	positive	positive	positive	NA	positive	positive	NA	NA	positive	42m negative
078	MRD- to MRD-	negative	negative	negative	negative	negative	negative	NA	NA	NA	
079	MRD+ to MRD+	positive	positive	positive	NA	NA	NA	NA	NA	NA	
080	MRD+ to MRD+	positive	positive	positive	NA	NA	NA	NA	NA	NA	
081	MRD- to MRD-	negative	negative	negative	NA	NA	negative	NA	NA	NA	
082	MRD+ to MRD-	positive	positive	negative	negative	NA	negative	negative	positive	positive	45m positive
083	MRD+ to MRD-	positive	negative	negative	negative	negative	negative	negative	negative	negative	42m negative
084	MRD+ to MRD-	positive	positive	NA	NA	negative	NA	NA	NA	NA	
085	MRD+ to MRD-	positive	negative	negative	NA	negative	NA	negative	negative	NA	
086	MRD+ to MRD-	positive	positive	positive	positive	positive	positive	positive	positive	positive	46m negative
087	MRD- to MRD+	negative	negative	positive	positive	positive	NA	NA	NA	NA	
088	MRD+ to MRD-	positive	positive	NA	NA	negative	NA	NA	NA	NA	
089	MRD+ to MRD+	positive	positive	NA	NA	NA	NA	NA	NA	NA	

090	MRD+ to MRD+	positive	NA	positive	positive	positive	positive	positive	NA	NA	
091	MRD+ to MRD-	positive	positive	NA	positive	negative	negative	NA	NA	negative	
092	MRD+ to MRD-	positive	negative	NA	NA	NA	NA	NA	NA	NA	
093	MRD- to MRD+	negative	positive	NA	positive	positive	NA	NA	NA	NA	
094	MRD+ to MRD-	positive	positive	positive	positive	positive	positive	positive	negative	negative	
095	MRD+ to MRD-	positive	positive	positive	positive	positive	positive	positive	positive	negative	44m negative
096	MRD+ to MRD-	positive	negative	negative	positive	positive	negative	positive	positive	positive	42m positive
097	MRD+ to MRD-	positive	positive	negative	negative	NA	positive	positive	positive	positive	49m positive
098	MRD- to MRD+	negative	positive	positive	NA	NA	NA	NA	NA	NA	
099	MRD- to MRD-	negative	negative	NA	NA	NA	NA	NA	NA	NA	
100	MRD+ to MRD-	positive	negative	positive	NA	NA	NA	NA	NA	NA	
101	MRD+ to MRD-	positive	negative	negative	positive	positive	negative	negative	NA	NA	
102	MRD- to MRD+	negative	negative	positive	NA	positive	positive	negative	positive	positive	37m positive
103	MRD- to MRD+	negative	negative	positive	positive	positive	positive	positive	negative	negative	
104	MRD- to MRD+	negative	negative	negative	negative	negative	negative	negative	positive	positive	5y positive
105	MRD- to MRD-	negative	negative	NA	NA	NA	negative	negative	negative	negative	57m negative
106	MRD- to MRD+	negative	positive	positive	NA	positive	positive	positive	positive	positive	64m positive
107	MRD- to MRD-	negative	negative	negative	negative	negative	NA	negative	NA	NA	
108	MRD+ to MRD-	positive	positive	negative	NA	positive	positive	positive	positive	positive	42m positive
109	MRD- to MRD-	negative	negative	NA	negative	negative	negative	negative	negative	negative	5y negative
110	MRD- to MRD+	negative	negative	negative	negative	negative	negative	negative	negative	positive	40m negative, 51m negative
111	MRD- to MRD+	negative	negative	negative	negative	negative	positive	NA	positive	NA	
112	MRD- to MRD+	negative	positive	positive	positive	NA	positive	positive	positive	NA	54m positive, 72m positive
113	MRD- to MRD-	negative	negative	negative	NA	negative	negative	negative	negative	negative	
114	MRD+ to MRD-	positive	negative	negative	NA	negative	negative	negative	NA	NA	
115	MRD+ to MRD+	positive	positive	positive	NA	positive	positive	NA	NA	NA	

116	MRD+ to MRD-	positive	negative	negative	negative	negative	negative	negative	NA	NA	44m negative
117	MRD+ to MRD+	positive	positive	positive	NA	NA	NA	NA	NA	NA	
118	MRD+ to MRD+	positive	positive	positive	NA	NA	NA	NA	NA	NA	
119	MRD+ to MRD+	positive	positive	positive	NA	positive	NA	NA	NA	NA	
120	MRD- to MRD-	negative	negative	NA	negative	negative	negative	negative	negative	negative	
121	MRD+ to MRD-	positive	negative	negative	NA	negative	negative	negative	NA	NA	48m negative
122	MRD+ to MRD+	positive	positive	positive	NA	positive	positive	NA	NA	NA	
123	MRD+ to MRD-	positive	negative	negative	positive	positive	positive	NA	NA	NA	
124	MRD+ to MRD+	positive	NA	positive	positive	positive	NA	NA	NA	positive	5y positive
125	MRD- to MRD-	negative	negative	negative	negative	negative	negative	negative	negative	negative	4y negative
126	MRD+ to MRD+	positive	positive	NA	NA	NA	NA	NA	NA	NA	
127	MRD- to MRD+	negative	positive	positive	NA	NA	NA	NA	NA	NA	
128	MRD- to MRD-	negative	negative	negative	NA	NA	NA	NA	NA	NA	
129	MRD- to MRD-	negative	negative	negative	negative	negative	negative	negative	negative	negative	4y negative
130	MRD+ to MRD-	positive	negative	NA	NA	NA	NA	NA	NA	NA	4y negative
131	MRD+ to MRD-	positive	positive	negative	negative	negative	negative	negative	negative	NA	52m negative
132	MRD+ to MRD-	positive	positive	positive	NA	negative	NA	positive	positive	positive	42m negative
133	MRD- to MRD-	negative	negative	negative	negative	negative	negative	negative	NA	negative	64m negative
134	MRD- to MRD+	negative	negative	negative	negative	negative	negative	positive	positive	positive	39m negative
135	MRD- to MRD-	negative	negative	negative	negative	negative	negative	NA	NA	NA	
136	MRD+ to MRD+	positive	positive	positive	NA	NA	NA	NA	NA	NA	
137	MRD- to MRD-	negative	negative	negative	negative	negative	negative	negative	negative	negative	4y negative
138	MRD+ to MRD-	positive	positive	negative	negative	positive	NA	NA	NA	NA	
139	MRD+ to MRD-	positive	positive	positive	positive	negative	negative	negative	negative	negative	5y positive, 5.5y negative
140	MRD- to MRD+	negative	negative	negative	negative	negative	negative	negative	positive	positive	40m positive
141	MRD+ to MRD-	positive	negative	negative	negative	negative	NA	negative	NA	NA	

142	MRD- to MRD+	negative	negative	negative	negative	negative	negative	negative	negative	negative	6.5y positive
143	MRD+ to MRD-	positive	negative	negative	NA	negative	negative	negative	positive	NA	
144	MRD+ to MRD-	positive	negative	negative	NA	NA	NA	NA	NA	NA	
145	MRD- to MRD-	negative	negative	negative	NA	negative	negative	negative	NA	NA	4y negative
146	MRD+ to MRD-	positive	negative	negative	negative	negative	negative	negative	negative	negative	48m negative
147	MRD+ to MRD+	positive	NA	positive	positive	positive	positive	NA	NA	NA	
148	MRD+ to MRD-	positive	positive	positive	negative	negative	negative	negative	negative	negative	66m positive
149	MRD- to MRD+	negative	negative	negative	positive	positive	positive	positive	positive	NA	
150	MRD+ to MRD+	positive	positive	positive	NA	NA	NA	NA	NA	NA	
151	MRD+ to MRD-	positive	negative	negative	negative	negative	positive	positive	NA	NA	
152	MRD+ to MRD-	positive	negative	negative	negative	negative	negative	negative	negative	NA	
153	MRD+ to MRD-	positive	positive	positive	negative	negative	negative	negative	negative	negative	
154	MRD+ to MRD-	positive	positive	positive	negative	negative	negative	negative	negative	negative	7.5y positive
155	MRD+ to MRD+	positive	positive	NA	NA	NA	NA	NA	NA	NA	
156	MRD+ to MRD-	positive	positive	negative	negative	negative	positive	negative	negative	positive	
157	MRD+ to MRD-	positive	negative	positive	NA	negative	positive	positive	positive	NA	
158	MRD+ to MRD-	positive	positive	positive	negative	negative	negative	negative	positive	positive	
159	MRD+ to MRD-	positive	negative	negative	NA	negative	NA	negative	positive	positive	60m positive
160	MRD- to MRD+	negative	positive	negative	negative	NA	NA	NA	NA	NA	
161	MRD- to MRD-	negative	negative	negative	NA	negative	NA	NA	NA	NA	
162	MRD+ to MRD-	positive	negative	negative	negative	negative	negative	negative	NA	NA	
163	MRD+ to MRD-	positive	positive	negative	NA	NA	NA	NA	NA	NA	
164	MRD+ to MRD-	positive	negative	NA	NA	NA	NA	NA	NA	NA	
165	MRD+ to MRD+	positive	positive	positive	positive	positive	NA	NA	NA	NA	
166	MRD+ to MRD-	positive	negative	negative	negative	negative	negative	negative	negative	positive	4y positive
167	MRD- to MRD+	negative	positive	negative	NA	negative	negative	negative	negative	negative	

168	MRD- to MRD-	negative	negative	negative	negative	negative	negative	negative	negative	negative	4y negative
169	MRD+ to MRD-	positive	positive	positive	NA	negative	NA	NA	NA	NA	
170	MRD- to MRD+	negative	negative	negative	NA	negative	negative	NA	NA	positive	48m positive
171	MRD- to MRD+	negative	negative	positive	NA	positive	NA	NA	NA	NA	
172	MRD+ to MRD+	positive	positive	positive	positive	positive	positive	positive	positive	positive	48m positive
173	MRD+ to MRD-	positive	positive	positive	positive	negative	negative	negative	negative	negative	42m negative
174	MRD+ to MRD+	positive	positive	positive	positive	positive	positive	positive	NA	positive	4y positive
175	MRD+ to MRD-	positive	negative	negative	negative	negative	negative	negative	positive	positive	39m negative
176	MRD+ to MRD+	positive	positive	positive	positive	positive	NA	positive	positive	positive	4y positive
177	MRD+ to MRD+	positive	positive	positive	positive	NA	NA	NA	NA	NA	
178	MRD- to MRD-	negative	negative	negative	negative	negative	negative	negative	negative	negative	
179	MRD- to MRD-	negative	negative	NA	NA	NA	NA	NA	NA	NA	
180	MRD+ to MRD-	positive	positive	positive	negative	negative	negative	positive	positive	positive	
181	MRD+ to MRD-	positive	positive	negative	negative	negative	negative	negative	NA	NA	
182	MRD- to MRD-	negative	negative	negative	negative	negative	NA	NA	NA	NA	
183	MRD+ to MRD-	positive	positive	negative	negative	negative	positive	positive	NA	NA	
184	MRD+ to MRD+	positive	positive	positive	positive	positive	positive	positive	positive	positive	48m positive
185	MRD+ to MRD-	positive	negative	negative	negative	negative	NA	NA	NA	NA	
186	MRD+ to MRD-	positive	positive	negative	negative	negative	positive	positive	NA	NA	
187	MRD+ to MRD-	positive	positive	positive	positive	negative	negative	negative	negative	negative	5y negative
188	MRD- to MRD-	negative	negative	negative	negative	negative	negative	NA	NA	NA	
189	MRD+ to MRD-	positive	positive	negative	negative	negative	negative	negative	NA	negative	5y negative
190	MRD+ to MRD-	positive	positive	positive	positive	positive	positive	positive	positive	negative	4.5y negative
191	MRD+ to MRD-	positive	negative	negative	negative	negative	negative	negative	NA	NA	
192	MRD+ to MRD-	positive	negative	negative	negative	negative	negative	NA	negative	positive	
193	MRD+ to MRD-	positive	positive	positive	negative	negative	negative	positive	positive	positive	40m positive

194	MRD+ to MRD-	positive	negative	negative	NA	NA	NA	NA	NA	NA	
195	MRD- to MRD-	negative	negative	negative	negative	negative	negative	negative	negative	NA	
196	MRD+ to MRD-	positive	positive	negative	negative	negative	negative	negative	negative	negative	67m positive
197	MRD- to MRD+	negative	negative	positive	positive	negative	negative	negative	negative	negative	4y positive
198	MRD+ to MRD-	positive	positive	negative	negative	negative	negative	negative	negative	positive	41m negative
199	MRD+ to MRD-	positive	negative	negative	negative	negative	negative	negative	positive	NA	
200	MRD+ to MRD-	positive	negative	negative	NA	negative	negative	negative	negative	negative	78m negative
201	MRD+ to MRD-	positive	negative	negative	negative	negative	negative	negative	negative	negative	9y negative
202	MRD- to MRD-	negative	negative	negative	NA	negative	negative	negative	negative	negative	7y negative
203	MRD- to MRD-	negative	negative	negative	negative	negative	negative	negative	NA	negative	4y negative
204	MRD- to MRD+	negative	negative	negative	NA	negative	negative	negative	positive	positive	
205	MRD- to MRD-	negative	negative	NA	NA	NA	NA	NA	NA	NA	
206	MRD- to MRD+	negative	negative	positive	positive	positive	positive	NA	positive	NA	
207	MRD- to MRD+	negative	negative	negative	negative	negative	negative	negative	positive	NA	
208	MRD+ to MRD-	positive	negative	positive	NA	NA	NA	NA	NA	NA	
209	MRD- to MRD+	negative	negative	positive	negative	negative	negative	negative	negative	NA	7.5y positive, 10.5y positive
210	MRD+ to MRD-	positive	negative	negative	negative	positive	NA	NA	NA	NA	
211	MRD+ to MRD-	positive	negative	negative	negative	negative	NA	negative	negative	NA	9y positive
212	MRD- to MRD-	negative	negative	negative	negative	negative	negative	negative	NA	negative	
213	MRD- to MRD+	negative	positive	positive	NA	NA	NA	NA	NA	NA	
214	MRD- to MRD+	negative	negative	negative	negative	negative	negative	negative	NA	positive	
215	MRD+ to MRD-	positive	positive	negative	negative	negative	negative	negative	negative	negative	5y positive
216	MRD+ to MRD-	positive	negative	negative	negative	negative	negative	negative	negative	NA	
217	MRD+ to MRD-	positive	negative	negative	negative	negative	negative	negative	negative	NA	
218	MRD+ to MRD-	positive	negative	negative	negative	negative	negative	negative	negative	negative	
219	MRD+ to MRD-	positive	negative	NA	negative	negative	negative	negative	negative	negative	

220	MRD- to MRD-	negative	negative	negative	negative	negative	negative	negative	NA	negative	
221	MRD+ to MRD+	positive	positive	positive	positive	NA	NA	NA	positive	positive	5.5y positive
222	MRD+ to MRD-	positive	positive	negative	negative	NA	NA	NA	NA	NA	
223	MRD+ to MRD-	positive	negative	negative	NA	NA	NA	NA	NA	NA	
224	MRD- to MRD+	negative	positive	positive	positive	positive	positive	negative	NA	negative	
225	MRD+ to MRD-	positive	negative	negative	negative	NA	negative	negative	NA	negative	9.5y negative
226	MRD- to MRD-	negative	negative	negative	negative	negative	NA	NA	NA	NA	
227	MRD+ to MRD-	positive	negative	negative	negative	negative	NA	positive	NA	NA	
228	MRD- to MRD+	negative	negative	negative	negative	negative	NA	negative	NA	NA	6y positive, 10y positive
229	MRD+ to MRD-	positive	negative	negative	NA	negative	negative	negative	NA	NA	
230	MRD- to MRD-	negative	negative	negative	NA	negative	NA	NA	NA	NA	
231	MRD- to MRD-	negative	negative	NA	NA	NA	NA	NA	NA	NA	
232	MRD+ to MRD-	positive	negative	negative	negative	negative	negative	negative	NA	negative	
233	MRD+ to MRD-	positive	negative	negative	negative	NA	NA	NA	NA	negative	6y negative
234	MRD+ to MRD-	positive	positive	negative	positive	NA	NA	NA	NA	NA	
235	MRD+ to MRD+	positive	NA	positive	NA	NA	NA	NA	NA	NA	
236	MRD- to MRD-	negative	negative	negative	negative	negative	negative	NA	NA	NA	
237	MRD+ to MRD-	positive	negative	negative	NA	negative	negative	NA	NA	NA	
238	MRD+ to MRD-	positive	negative	negative	NA	negative	negative	NA	NA	NA	
239	MRD- to MRD-	negative	negative	NA	NA	NA	NA	NA	NA	NA	
240	MRD+ to MRD-	positive	positive	positive	positive	negative	negative	negative	NA	NA	
241	MRD+ to MRD-	positive	negative	positive	negative	NA	NA	NA	NA	NA	
242	MRD+ to MRD-	positive	negative	negative	negative	negative	negative	NA	NA	NA	
243	MRD- to MRD-	negative	negative	negative	negative	negative	negative	negative	NA	NA	
244	MRD+ to MRD-	positive	negative	negative	negative	negative	negative	NA	NA	NA	
245	MRD+ to MRD-	positive	negative	negative	negative	negative	negative	negative	NA	NA	

246	MRD+ to MRD-	positive	negative	negative	positive	positive	negative	NA	NA	NA	
247	MRD- to MRD-	negative	negative	negative	negative	negative	negative	NA	NA	NA	
248	MRD- to MRD-	negative	negative	NA	negative	negative	NA	NA	NA	NA	
249	MRD+ to MRD-	positive	negative	negative	NA	negative	negative	NA	NA	NA	
250	MRD- to MRD-	negative	negative	NA	NA	negative	NA	negative	NA	NA	
251	MRD- to MRD+	negative	negative	NA	NA	NA	positive	NA	NA	NA	
252	MRD+ to MRD-	positive	positive	positive	NA	positive	negative	NA	NA	NA	
253	MRD+ to MRD-	positive	positive	negative	negative	negative	NA	NA	NA	NA	
254	MRD- to MRD-	negative	negative	negative	NA	negative	negative	negative	NA	NA	
255	MRD- to MRD-	negative	negative	NA	NA	negative	NA	NA	NA	NA	
256	MRD+ to MRD-	positive	negative	negative	negative	negative	NA	positive	NA	NA	
257	MRD+ to MRD+	positive	positive	positive	NA	positive	NA	positive	NA	NA	
258	MRD- to MRD-	negative	negative	NA	NA	negative	NA	NA	NA	NA	
259	MRD- to MRD+	negative	negative	negative	negative	NA	positive	NA	NA	NA	
260	MRD- to MRD-	negative	negative	negative	negative	negative	NA	negative	NA	NA	
261	MRD+ to MRD-	positive	positive	positive	positive	positive	negative	negative	NA	NA	
262	MRD+ to MRD-	positive	positive	positive	positive	positive	positive	positive	negative	NA	
263	MRD- to MRD-	negative	negative	NA	negative	negative	negative	negative	negative	NA	
264	MRD+ to MRD-	positive	positive	negative	negative	positive	negative	positive	positive	NA	
265	MRD- to MRD-	negative	negative	negative	negative	negative	negative	negative	negative	NA	
266	MRD+ to MRD+	positive	positive	positive	positive	positive	positive	NA	NA	NA	
267	MRD- to MRD-	negative	negative	negative	negative	negative	negative	negative	negative	NA	
268	MRD+ to MRD+	positive	positive	NA	NA	NA	NA	NA	NA	NA	
269	MRD- to MRD-	negative	negative	negative	negative	negative	negative	negative	negative	negative	5y negative
270	MRD- to MRD-	negative	NA	NA	NA	negative	NA	NA	NA	NA	
271	MRD+ to MRD-	positive	positive	positive	NA	negative	NA	NA	NA	NA	

272	MRD+ to MRD+	positive	positive	positive	positive	positive	positive	NA	NA	NA	
273	MRD+ to MRD+	positive	positive	positive	positive	positive	NA	NA	NA	NA	
274	MRD+ to MRD-	positive	positive	negative	negative	negative	negative	negative	NA	NA	4.5y positive
275	MRD- to MRD-	negative	negative	negative	negative	negative	negative	negative	negative	negative	46m negative
276	MRD- to MRD-	negative	negative	negative	negative	negative	negative	negative	negative	negative	
277	MRD+ to MRD-	positive	negative	negative	NA	negative	positive	positive	NA	NA	
278	MRD+ to MRD-	positive	negative	negative	negative	negative	NA	NA	NA	NA	
279	MRD+ to MRD-	positive	negative	negative	negative	NA	NA	NA	NA	NA	
280	MRD- to MRD+	negative	positive	positive	NA	positive	NA	NA	NA	NA	
281	MRD- to MRD-	negative	negative	NA	NA	negative	negative	negative	negative	negative	
282	MRD+ to MRD-	positive	positive	negative	negative	negative	negative	negative	negative	negative	
283	MRD+ to MRD+	positive	positive	positive	positive	positive	positive	positive	positive	NA	
284	MRD+ to MRD-	positive	negative	negative	negative	negative	negative	NA	NA	NA	
285	MRD+ to MRD+	positive	positive	positive	NA	positive	positive	NA	NA	NA	
286	MRD- to MRD+	negative	positive	positive	NA	NA	NA	NA	NA	NA	
287	MRD- to MRD-	negative	negative	negative	NA	negative	negative	NA	NA	NA	
288	MRD- to MRD-	negative	negative	negative	negative	negative	NA	NA	NA	NA	
289	MRD+ to MRD-	positive	positive	positive	positive	negative	negative	NA	NA	NA	
290	MRD+ to MRD-	positive	negative	negative	negative	negative	negative	NA	NA	NA	
291	MRD+ to MRD-	positive	negative	negative	negative	negative	negative	NA	NA	NA	
292	MRD+ to MRD-	positive	negative	NA	negative	negative	negative	NA	NA	NA	
293	MRD+ to MRD-	positive	negative	positive	NA	negative	negative	NA	NA	NA	
294	MRD+ to MRD-	positive	negative	negative	negative	negative	NA	NA	NA	NA	
295	MRD+ to MRD-	positive	negative	negative	negative	negative	NA	NA	NA	NA	
296	MRD- to MRD-	negative	negative	NA	NA	NA	NA	NA	NA	NA	
297	MRD- to MRD-	negative	negative	negative	negative	negative	NA	NA	NA	NA	

298	MRD+ to MRD-	positive	nega-tive	nega-tive	nega-tive	nega-tive	NA	NA	NA	NA
299	MRD+ to MRD-	positive	nega-tive	nega-tive	nega-tive	NA	NA	NA	NA	NA
300	MRD+ to MRD+	positive	positive	positive	NA	NA	NA	NA	NA	NA
301	MRD- to MRD-	nega-tive	nega-tive	nega-tive	nega-tive	nega-tive	NA	NA	NA	NA
302	MRD+ to MRD-	positive	positive	nega-tive	NA	NA	NA	NA	NA	NA
303	MRD+ to MRD-	positive	nega-tive	nega-tive	nega-tive	nega-tive	NA	NA	NA	NA
304	MRD+ to MRD-	positive	nega-tive	NA	nega-tive	NA	NA	NA	NA	NA
305	MRD- to MRD-	nega-tive	nega-tive	NA	NA	NA	NA	NA	NA	NA
306	MRD- to MRD-	nega-tive	nega-tive	nega-tive	nega-tive	nega-tive	NA	NA	NA	NA
307	MRD+ to MRD-	positive	positive	nega-tive	nega-tive	NA	NA	NA	NA	NA
308	MRD- to MRD-	nega-tive	NA	nega-tive	nega-tive	nega-tive	NA	NA	NA	NA
309	MRD- to MRD-	nega-tive	nega-tive	nega-tive	NA	nega-tive	NA	NA	NA	NA
310	MRD- to MRD-	nega-tive	nega-tive	NA	nega-tive	nega-tive	NA	NA	NA	NA
311	MRD+ to MRD+	positive	positive	positive	positive	NA	NA	NA	NA	NA
312	MRD+ to MRD-	positive	nega-tive	NA	NA	NA	NA	NA	NA	NA
313	MRD- to MRD+	nega-tive	positive	nega-tive	nega-tive	nega-tive	NA	NA	NA	NA
314	MRD- to MRD+	nega-tive	nega-tive	positive	NA	positive	NA	NA	NA	NA
315	MRD+ to MRD-	positive	nega-tive	NA	nega-tive	NA	NA	NA	NA	NA
316	MRD+ to MRD+	positive	positive	positive	NA	NA	NA	NA	NA	NA
317	MRD+ to MRD+	positive	positive	NA	NA	NA	NA	NA	NA	NA
318	MRD+ to MRD-	positive	positive	positive	nega-tive	NA	NA	NA	NA	NA
319	MRD- to MRD-	nega-tive	nega-tive	nega-tive	nega-tive	NA	NA	NA	NA	NA
320	MRD+ to MRD-	positive	nega-tive	NA	NA	NA	NA	NA	NA	NA
321	MRD- to MRD-	nega-tive	nega-tive	nega-tive	nega-tive	NA	NA	NA	NA	NA
322	MRD- to MRD-	nega-tive	nega-tive	nega-tive	nega-tive	NA	NA	NA	NA	NA
323	MRD+ to MRD+	positive	positive	positive	NA	NA	NA	NA	NA	NA

324	MRD+ to MRD-	positive	negative	NA	NA	NA	NA	NA	NA	NA
325	MRD+ to MRD-	positive	negative	NA	NA	NA	NA	NA	NA	NA
326	MRD+ to MRD-	positive	negative	negative	NA	negative	NA	NA	NA	NA
327	MRD+ to MRD+	positive	positive	positive	NA	NA	NA	NA	NA	NA
328	MRD- to MRD-	negative	negative	NA	NA	NA	NA	NA	NA	NA
329	MRD- to MRD-	negative	negative	NA	NA	NA	NA	NA	NA	NA
330	MRD- to MRD-	negative	negative	NA	NA	NA	NA	NA	NA	NA
331	MRD+ to MRD-	positive	negative	NA	NA	NA	NA	NA	NA	NA
332	MRD- to MRD-	negative	negative	NA	NA	NA	NA	NA	NA	NA
333	MRD- to MRD-	negative	negative	NA	NA	NA	NA	NA	NA	NA
334	MRD- to MRD-	negative	negative	negative	NA	NA	NA	NA	NA	NA
335	MRD- to MRD-	negative	negative	negative	NA	NA	NA	NA	NA	NA

Table S2. Summary table of MRD assessment frequency.

Category	Mean (number)	Median (number)	Minimum (number)	Maximum (number)	Range
MRD- to MRD-	5.71	6	2	10	8
MRD- to MRD+	7.05	8	3	11	8
MRD+ to MRD-	6.68	7	2	11	9
MRD+ to MRD+	4.76	4	2	10	8

Note: Statistics were based on MRD assessments documented in Supplementary Table S1.

Table S3. Median Overall Survival (OS) and Progression-Free Survival (PFS) according to different MRD dynamic patterns.

Group	OS median	PFS median
MRD status		
MRD- to MRD-	not reached	not reached
MRD- to MRD+	105	50
MRD+ to MRD-	131	71
MRD+ to MRD+	87	38
MRD- to MRD+		
MRD- to MRD+ early	116	44
MRD- to MRD+ late	84	50
MRD+ to MRD+		
MRD+ to MRD- early	133	81
MRD+ to MRD- late	119	58
MRD+ to MRD+		
MRD+ to MRD+ decreasing	106	59
MRD+ to MRD+ stable or increasing	50	20

Table S4. Univariate and multivariate analyses of OS and PFS using different MRD statuses as reference.

Category	Univariate			Multivariate		
	HR	95%CI	*P*-value	HR	95%CI	*P*-value
			OS			
MRD status						
MRD- to MRD-	0.262	0.126 - 0.547	<0.001	0.255	0.116 - 0.558	<0.001
MRD- to MRD+	1			1		
MRD+ to MRD-	0.528	0.314 - 0.886	0.016	0.503	0.295 - 0.858	0.012
MRD+ to MRD+	1.317	0.717 - 2.418	0.374	1.214	0.631 - 2.335	0.562
MRD status						
MRD- to MRD-	0.497	0.257 - 0.962	0.038	0.507	0.247 - 1.039	0.063
MRD- to MRD+	1.894	1.128 - 3.181	0.016	1.989	1.165 - 3.395	0.012
MRD+ to MRD-	1			1		
MRD+ to MRD+	2.495	1.482 - 4.199	0.001	2.413	1.347 - 4.325	0.003
MRD status						
MRD- to MRD-	0.199	0.095 - 0.417	<0.001	0.210	0.095 - 0.465	<0.001
MRD- to MRD+	0.759	0.414 - 1.394	0.374	0.824	0.428 - 1.586	0.562
MRD+ to MRD-	0.401	0.238 - 0.675	0.001	0.414	0.231 - 0.743	0.003
MRD+ to MRD+	1			1		
			PFS			
MRD status						
MRD- to MRD-	0.226	0.127 - 0.399	<0.001	0.226	0.125 - 0.408	<0.001
MRD- to MRD+	1			1		
MRD+ to MRD-	0.527	0.349 - 0.794	0.002	0.523	0.345 - 0.793	0.002
MRD+ to MRD+	0.973	0.582 - 1.626	0.916	0.993	0.567 - 1.742	0.981
MRD status						
MRD- to MRD-	0.428	0.260 - 0.706	0.001	0.431	0.256 - 0.726	0.002
MRD- to MRD+	1.899	1.260 - 2.863	0.002	1.911	1.261 - 2.896	0.002
MRD+ to MRD-	1			1		
MRD+ to MRD+	1.848	1.195 - 2.858	0.006	1.898	1.169 - 3.081	0.010
MRD status						
MRD- to MRD-	0.232	0.129 - 0.418	<0.001	0.227	0.118 - 0.436	<0.001
MRD- to MRD+	1.028	0.615 - 1.718	0.916	1.007	0.574 - 1.765	0.981
MRD+ to MRD-	0.541	0.350 - 0.837	0.006	0.527	0.325 - 0.855	0.010
MRD+ to MRD+	1			1		

OS adjusted for R-ISS staging, hemoglobin, serum LDH levels, HRCAs, cytogenetic abnormality, and maintenance treatment; PFS adjusted for age, R-ISS staging, HRCAs, cytogenetic abnormality, maintenance treatment, and response at ASCT.

Table S5. Proportional hazards assumption testing for MRD dynamic groups in the Cox regression model.

Comparison	Spearman correlation (r)	*P*-value	*P*-value for interaction term
	OS		
MRD- to MRD+ vs. MRD- to MRD-	-0.387	<0.001	0.809
MRD+ to MRD- vs. MRD- to MRD-	-0.383	<0.001	0.774
MRD+ to MRD+ vs. MRD- to MRD-	-0.019	0.854	-*
	PFS		
MRD- to MRD+ vs. MRD- to MRD-	0.122	0.127	-**
MRD+ to MRD- vs. MRD- to MRD-	-0.341	<0.001	0.437
MRD+ to MRD+ vs. MRD- to MRD-	0.227	0.004	0.540

Spearman's rank correlation test between partial residuals and survival time (OS or PFS months). *P*-value for the interaction term between the dummy variable and time in the extended Cox model. * The PH assumption was satisfied for the persistent MRD+ group in the OS model, so no interaction term was introduced. ** The PH assumption was satisfied for the MRD- to MRD+ group in the PFS model, so no interaction term was introduced. OS adjusted for R-ISS staging, hemoglobin, serum LDH levels, HRCAs, cytogenetic abnormality, and maintenance treatment; PFS adjusted for age, R-ISS staging, HRCAs, cytogenetic abnormality, maintenance treatment, and response at ASCT.
